# Limb Compression Therapy and Chemotherapy‐Induced Peripheral Neuropathy in Women With Gynecologic Cancers: A Prospective Self‐Controlled Study(NEURO‐GLOVE Trial)

**DOI:** 10.1002/ijc.70601

**Published:** 2026-06-18

**Authors:** Kadriye Başkurt, Galip Can Uyar, Enes Yeşilbaş, Fatma Zehra Altunç, Damla Erimhan Çevik, İsmet Murat Melek, Yasemin Eren, Ömür Berna Çakmak Öksüzoğlu, Kadriye Bir Yücel, Antonio Di Meglio, Isabelle Ray‐Coquard, Osman Sütcüoğlu

**Affiliations:** ^1^ Department of Medical Oncology Etlik City Hospital Ankara Turkey; ^2^ Department of Neurology Etlik City Hospital Ankara Turkey; ^3^ Université Paris‐Saclay, Gustave Roussy, Inserm, CESP Villejuif France; ^4^ Department of Medical Oncology Centre Léon Bérard Lyon France; ^5^ Department of Medical Oncology Gazi University Faculty of Medicine Ankara Turkey

**Keywords:** chemotherapy‐induced peripheral neuropathy, gynecologic neoplasms, mechanical compression, paclitaxel, peripheral nerve disorders

## Abstract

Chemotherapy‐induced peripheral neuropathy (CIPN) is a dose‐limiting toxicity of taxane‐based chemotherapy with limited preventive options. We evaluated whether dual‐site mechanical compression during paclitaxel infusion reduces CIPN in women with gynecologic cancers. In this prospective, intraindividual, self‐controlled study, patients receiving carboplatin–paclitaxel underwent compression of the nondominant hand (two surgical gloves) and ipsilateral lower limb (Class II stocking), while contralateral limbs served as controls. CIPN was assessed using CTCAE v5.0 and EORTC QLQ‐CIPN20 at baseline, Cycle 3, end of treatment (EOT), and 3‐ and 6‐month follow‐up. Among 76 patients (median age, 61 years), moderate‐to‐severe CIPN was observed less frequently in compression limbs than in control limbs at Cycle 3 (35.5% vs. 53.9%; *p* = 0.001), EOT (59.2% vs. 69.7%; *p* = 0.021), 3 months (28.9% vs. 52.6%; *p* = 0.015), and 6 months (20.3% vs. 33.8%; *p* = 0.002). Repeated‐measures analyzes showed significant time‐by‐limb interactions for sensory (*p* = 0.003), lower‐extremity sensory (*p* = 0.018), and motor domains (*p* = 0.041). Sensory CIPN20 scores were lower in compression limbs in the upper extremities from EOT onward (7.5 vs. 10.2; *p* < 0.001) and in the lower extremities at 3‐month (8.7 vs. 10.7; *p* < 0.001) and 6‐month follow‐up (7.0 vs. 10.0; *p* < 0.001). EORTC QLQ‐CIPN20 sensory scores identified grade ≥ 2 neuropathy with AUC values > 0.92 at all time points. Dual‐site mechanical compression was associated with reduced incidence and persistence of CIPN. As a low‐cost and scalable intervention, this strategy may improve treatment tolerability and survivorship outcomes.

**Trial Registration:** ClinicalTrials.gov identifier: NCT07105553

## Abstract (highlights)

What's new?

With no effective drug‐based prevention strategies available for chemotherapy‐induced peripheral neuropathy (CIPN), research in neuroprotective approaches has grown. Of particular interest is mechanical compression, which may protect nerves by temporarily reducing blood flow during treatment. The authors here conducted a prospective trial in women with gynecologic cancers, applying mechanical compression to one hand and leg during treatment while using the opposite limbs as controls. Compression‐treated limbs consistently experienced lower rates and severity of neuropathy during treatment and up to six months afterward. As a low‐cost, scalable intervention, dual‐site mechanical compression can potentially improve treatment tolerability and reduce long‐term nerve damage.

## Graphical abstract

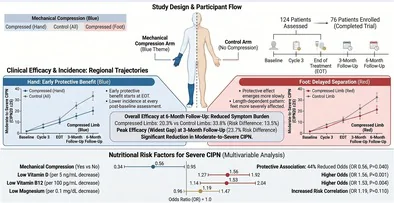

**Abbreviations**
BMI: body mass index
CA‐125: cancer antigen 125
CIPN: chemotherapy‐induced peripheral neuropathy
CTCAE: common terminology criteria for adverse events
ECOG: Eastern cooperative oncology group
EMG: electromyography
EORTC QLQ‐CIPN20: European Organisation for Research and Treatment of Cancer Quality of Life Questionnaire–Chemotherapy‐Induced Peripheral Neuropathy 20‐item scale
EOT: end of treatment
GEE: generalized estimating equation
LDH: lactate dehydrogenase
mCCI: modified Charlson Comorbidity Index
RF: random forest

## 1 Introduction

Gynecologic malignancies, including ovarian and endometrial cancers, impose a substantial global health burden, with more than 700,000 new diagnoses annually. Paclitaxel‐based chemotherapy remains a cornerstone of treatment for ovarian and endometrial cancers [1, 2, 3, 4]. Despite its efficacy, paclitaxel is frequently associated with chemotherapy‐induced peripheral neuropathy (CIPN), a common and potentially persistent toxicity that impairs physical function and quality of life and often necessitates dose reduction or premature treatment discontinuation [5, 6, 7, 8]. Clinically significant CIPN develops in approximately 30% to 60% of patients receiving paclitaxel, and symptoms may persist long after treatment completion [9, 10, 11].

To date, no pharmacologic intervention has demonstrated consistent, high‐level evidence for the prevention of CIPN. Clinical trials of antioxidants, neurotrophic agents, anticonvulsants, and vitamin‐based therapies have shown limited or inconsistent benefit, and current ASCO and ESMO guidelines do not recommend any pharmacologic agent for standard CIPN prophylaxis [9, 10]. Consequently, attention has increasingly shifted toward non‐pharmacologic neuroprotective strategies. Regional cooling and mechanical compression are hypothesized to transiently reduce limb microvascular perfusion, thereby limiting taxane delivery to peripheral nerves [12, 13, 14]. Physiological studies support this concept, suggesting that external compression may modulate blood flow and microvascular perfusion [15].

However, clinical evidence supporting mechanical compression for CIPN prevention remains inconsistent. Some studies, including the POLAR randomized clinical trial, have suggested potential reductions in taxane‐induced neuropathy with surgical glove compression, while small feasibility studies indicate that combined cooling or compression may be tolerable and possibly beneficial [13, 16]. In contrast, other prospective investigations, including a double‐blind phase II trial, have reported minimal or no preventive effect [17]. Moreover, most available data derive from breast cancer cohorts and focus predominantly on hand‐only interventions, despite the substantial burden of lower‐extremity neuropathy in patients receiving taxane‐based chemotherapy [17, 18]. Reflecting these limitations, current ASCO and ESMO guidelines consider the evidence for mechanical compression and other non‐pharmacologic interventions to be insufficient for routine clinical use [9, 10].

Given the absence of established CIPN preventive strategies in gynecologic oncology, practical, scalable, and low‐cost interventions addressing both upper and lower extremities are needed. In this prospective self‐controlled study, we evaluated whether dual‐site mechanical compression using surgical gloves and ipsilateral lower limb compression stockings during paclitaxel infusion was associated with a reduced incidence and severity of CIPN in women with gynecologic cancers.

## 2 Methods

### 2.1 Study Design, Patients, and Treatment Details

This prospective self‐controlled study enrolled women with histologically confirmed ovarian or endometrial cancer who were scheduled to receive standard three‐weekly carboplatin–paclitaxel chemotherapy at Etlik City Hospital, a high‐volume tertiary care academic center, between December 2023 and December 2024. Eligible patients were ≥ 18 years of age, had no history of severe cardiovascular or peripheral vascular disease, and planned to receive six cycles of adjuvant chemotherapy after surgery or six cycles of systemic therapy for metastatic disease. Reporting followed the Transparent Reporting of Evaluations with Nonrandomized Designs (TREND) statement. The TREND Statement Checklist can be found in Appendix 1 in Supporting Information.

To ensure a homogeneous chemotherapy protocol and uniform neurotoxic exposure, patients receiving weekly paclitaxel, docetaxel‐based chemotherapy, or prior neurotoxic agents were excluded. Additionally, patients who had received neoadjuvant chemotherapy before surgery were excluded to avoid heterogeneity in cumulative neurotoxic exposure. To establish a neuropathy‐free cohort at study entry, patients with any evidence of preexisting peripheral neuropathy, including common terminology criteria for adverse events (CTCAE) version 5.0 [19] grade ≥ 1 at baseline or European Organisation for Research and Treatment of Cancer Quality of Life Questionnaire–Chemotherapy‐Induced Peripheral Neuropathy 20‐item scale (EORTC QLQ‐CIPN20)‐reported neuropathic symptoms, were excluded. Additional exclusion criteria included neurologic disorders unrelated to chemotherapy, diabetes with neuropathic involvement, and use of medications that could mask or modify neuropathic symptoms (e.g., gabapentinoids, serotonin–norepinephrine reuptake inhibitors). All participants underwent baseline EORTC QLQ‐CIPN20 [20] assessment confirming absence of neuropathy symptoms before enrollment.

Paclitaxel was administered intravenously at 175 mg/m^2^ every 21 days in combination with carboplatin at an AUC of 5. Chemotherapy dose reductions, delays, or early discontinuation were made at the discretion of treating oncologists based on toxicity and clinical status.

### 2.2 Intervention

During each paclitaxel infusion, mechanical compression was applied to the nondominant upper limb and the ipsilateral lower limb. Patients wore two tight surgical gloves on the nondominant hand and a Class II (20–30 mmHg), knee‐high graduated compression stocking (RAL‐GZ 387 certified) on the same‐side lower‐extremity. Hand circumference at the metacarpophalangeal joint was measured to determine glove size, and two gloves one size smaller than the measured fit were used to ensure adequate compression (Figure S1) [13, 21]. The nondominant upper limb and ipsilateral lower‐extremity were selected for compression to ensure unimpeded venous access for chemotherapy administration in the dominant arm and to facilitate consistent protocol application across patients. This approach is consistent with prior compression trials, including the POLAR randomized clinical trial [13]. Latex‐free gloves were used to avoid contact sensitivity. Peripheral venous access was successfully established in all patients, allowing for consistent application of the compression protocol to the nondominant upper limb as planned.

Compression began 30 min before paclitaxel infusion, continued throughout chemotherapy, and was maintained 30 min after completion. The contralateral upper and lower limbs received no intervention and served as intra‐patient controls. Compression tolerance, local discomfort, and skin reactions were assessed at each infusion. No clinically significant adverse events related to compression were observed during the study period. Specifically, no patient required early removal of the compression devices, no skin reactions or contact sensitivity were recorded, and no compression‐related complications required protocol modification. Adherence to the compression protocol was monitored by the infusion nurse at each chemotherapy session, who confirmed the correct application and duration of both the surgical gloves and compression stocking throughout the infusion period. Patients who developed CTCAE grade ≥ 2 neuropathy were referred to neurology, and electromyography (EMG) was performed when clinically indicated.

### 2.3 Clinical Assessment

Neuropathy assessments were performed at five predefined time points: baseline, after Cycle 3, at end of treatment (EOT), and at 3‐month and 6‐month posttreatment follow‐up. At each evaluation, a structured neurologic examination by an oncology specialist assessed sensory and motor symptoms, functional impairment, and neurologic signs. CIPN was graded using CTCAE v5.0, with grade ≥ 2 defined as clinically significant neuropathy.

Patient‐reported neuropathy symptoms were assessed using the EORTC QLQ‐CIPN20 instrument, which evaluates sensory, motor, and autonomic domains [20]. EORTC QLQ‐CIPN20 sensory, motor, autonomic, and total scores were computed and transformed to a 0–100 scale following EORTC scoring guidelines, with higher scores indicating more severe symptoms. Clinically significant neuropathy was defined as a total EORTC QLQ‐CIPN20 score ≥ 25, a threshold applied consistently across all time points. Additional details regarding score computation and subscale derivation are provided in supplementary methods.

CTCAE neuropathy grading was performed by the treatment medical oncologist team (K.B., G.C.U., and E.Y.) and consulting neurologists (F.Z.A. and D.E.Ç.), with supervisory oversight by senior neurologists (İ.M.M. and Y.E.) and senior medical oncologists (Ö.B.Ç.Ö. and O.S.) at each assessment. Baseline demographic, clinical, and laboratory variables, including age, Body Mass Index (BMI), Eastern Cooperative Oncology Group (ECOG) performance status, modified Charlson Comorbidity Index (mCCI) [22], weight change during chemotherapy, HbA1c, lactate dehydrogenase (LDH), vitamin D, vitamin B12, CRP/albumin ratio (CAR), magnesium, cancer antigen‐125 (CA‐125), and electrolytes, were recorded. Cumulative paclitaxel dose (mg/m^2^) was calculated as the total amount received across cycles. Concomitant metformin and statin use and BRCA mutation status were also documented. Patients who developed CTCAE grade ≥ 2 neuropathy were referred to neurology for clinical evaluation. EMG was performed in patients for whom the consulting neurologist recommended further electrophysiological assessment and who provided consent for the procedure. For patients evaluated by EMG, agreement between EMG findings and both CTCAE and EORTC QLQ‐CIPN20 classifications was assessed. Participants who missed follow‐up assessments were not replaced, and all analyzes used complete‐case data. Missing data were limited to the 6‐month follow‐up time point, where assessments were unavailable for two patients due to death. No missing data occurred at baseline, Cycle 3, EOT, or 3‐month follow‐up.

### 2.4 Statistical Analysis

A priori sample size estimation was performed using G*Power (version 3.1.9.7) for a McNemar test (*z*‐test, two‐tailed, *α* = 0.05, power = 0.80). Based on an anticipated 20% absolute reduction in grade ≥ 2 CIPN incidence with compression, derived from prior compression trial data, a minimum sample size of 72 patients was estimated to achieve 80% power. The enrolled sample of 76 patients exceeded this threshold, yielding an achieved power of 82.1%.

Statistical analyzes accounted for the self‐controlled, paired‐limb study design. Continuous outcomes were compared using paired *t*‐tests or Wilcoxon signed‐rank tests, as appropriate. Paired categorical outcomes, including clinically significant neuropathy defined as CTCAE grade ≥ 2 and EORTC QLQ‐CIPN20 total score ≥ 25, were compared using McNemar tests. Longitudinal changes in EORTC QLQ‐CIPN20 sensory, motor, and total scores were evaluated using repeated‐measures analysis of variance with limb (compression vs. control) and time as within‐patient factors.

To estimate the independent association between mechanical compression and moderate‐to‐severe neuropathy while accounting for within‐patient correlation, generalized estimating equation models with a binomial distribution and logit link were fitted, specifying patient identifier as the clustering variable and an exchangeable working correlation structure. Models were adjusted for demographic, clinical, and laboratory covariates. Covariates included in the GEE model were selected based on clinical relevance and prior literature on CIPN risk factors. Variance inflation factors and tolerance values were examined to assess multicollinearity; all variables had VIF values below five and tolerance values above 0.30.

Additional statistical methods, including agreement analyzes, discriminative performance analyzes, electrophysiologic correlations, effect size estimation, and exploratory machine learning analyzes, are described in the supplementary methods. Statistical analyzes were performed using SPSS version 25.0 (IBM Corp, Armonk, NY, USA) and R version 4.5.2 (R Foundation for Statistical Computing, Vienna, Austria).

## 3 Results

### 3.1 Patient Characteristics and Study Flow

Baseline demographic, clinical, laboratory, and treatment characteristics of the 76 enrolled patients are shown in Table 1. Median age was 61 years (IQR, 53–67). Median follow‐up was 14.6 months (IQR, 12.1–19.6). Median body weight was 73 kg (IQR, 63–85), and median height was 1.56 m (IQR, 1.52–1.60). Overall, 74 patients (97.4%) completed all six planned cycles of carboplatin–paclitaxel chemotherapy; paclitaxel was permanently discontinued after Cycle 4 in two patients (2.6%) due to CIPN, and both completed six cycles of carboplatin. Further dose modifications are detailed in Table 1; paclitaxel dose reductions and delays due to CIPN were recorded in 13 (17.1%) and 4 (5.3%) patients, respectively. Although these modifications altered cumulative exposure for both limbs, dose modification decisions were based on the patient's overall clinical neuropathy assessment. In all affected cases, clinically significant neuropathy was present in the control limb at the time of modification, while the compression limb demonstrated lower neuropathy grades at the same assessment. This pattern should be interpreted cautiously, as it may reflect overall neuropathy burden rather than true biological asymmetry between limbs.

**TABLE 1:** Baseline demographic, clinical, and treatment characteristics.

Parameters (*n* = 76)	*n* (%) or median (IQR)	Parameters (*n* = 76)	*n* (%) or median (IQR)
Age at diagnosis (years)		Treatment interruption	
< 65	47 (61.8)	None	50 (65.8)
≥ 65	29 (38.2)	Due to CIPN	2 (2.6)
Dominant hand and foot		Due to cytopenia	12 (15.8)
Right	33 (43.4)	Others	12 (15.8)
Left	43 (56.6)	Treatment response at Cycle 3	
Smoking history		Stable	76 (100.0)
Absent	52 (68.4)	PD	0 (0.0)
Present	24 (31.6)	MSI status	
Alcohol history		MSS	72 (94.7)
Absent	71 (93.4)	MSI‐H	4 (5.3)
Present	5 (6.6)	Recurrence during follow‐up (DFS)	
ECOG‐PS		Absent	60 (78.9)
0–1	56 (73.7)	Present	16 (21.1)
2	20 (26.3)	Chemotherapy delay	
Primary diagnosis		None	36 (47.4)
Endometrial cancer	43 (56.6)	Due to CIPN	4 (5.3)
Ovarian cancer	33 (43.4)	Due to cytopenia	26 (34.2)
Disease stage		Others	10 (13.2)
I–II	35 (46.1)	Baseline vitamin B12 supplementation within past month	
III–IV	41 (53.9)	Absent	60 (78.9)
Statin use		Present	16 (21.1)
Absent	67 (88.2)	Baseline vitamin D supplementation within past month	
Present	9 (11.8)	Absent	56 (73.7)
Baseline HbA1c (%)		Present	20 (26.3)
< 6.5	60 (78.9)	Baseline CA‐125 (U/mL)	
≥ 6.5	16 (21.1)	< 35	33 (43.4)
BRCA status		≥ 35	43 (56.6)
Wild type	66 (86.8)	Paclitaxel dose reduction	
Mutant	10 (13.2)	None	51 (67.1)
Weight loss ≥ 10% (end of treatment)		Due to CIPN	13 (17.1)
Absent	61 (80.3)	Due to cytopenia	7 (9.2)
Present	15 (19.7)	Due to weight loss	5 (6.6)
Death (any cause)		Treatment response at EOT	
Absent	74 (97.4)	Non‐PD	74 (97.4)
Present	2 (2.6)	PD	2 (2.6)
Paclitaxel discontinuation^a^		Maintenance olaparib	
Absent	74 (97.4)	Absent	71 (93.4)
Present	2 (2.6)	Present	5 (6.6)
BMI (kg/m^2^)	30.4 (26.5–34.5)	EMG‐confirmed peripheral neuropathy at EOT	
C‐reactive protein, mg/L	7.1 (3.9–16.9)	Absent	14 (29.8)
Serum albumin, g/L	42.5 (39.3–44.0)	Present	33 (70.2)
CAR	0.18 (0.09–0.42)	Not assessed	29 (38.2)
mCCI	7 (4–9)	Serum magnesium, mg/dL	1.90 (1.60–2.04)
Vitamin D, ng/mL	13.0 (7.3–22.0)	Vitamin B12, pg/mL	305 (219–403)
Cumulative taxane dose, mg/m^2^	1680 (1500–1855)	LDH, U/L	197 (176–223)

*Note:* Continuous variables are presented as median (interquartile range) due to non‐normal distributions. Categorical variables are presented as number (percentage). CRP and albumin are reported descriptively and were not included in regression analyzes because the CRP‐to‐albumin ratio (CAR) was used as the composite inflammatory marker.

Abbreviations: BMI, body mass index; BRCA, breast cancer susceptibility gene; CA‐125, cancer antigen 125; CAR, C‐reactive protein‐to‐albumin ratio; CIPN, chemotherapy‐induced peripheral neuropathy; DFS, disease‐free survival; ECOG‐PS, Eastern Cooperative Oncology Group performance status; EMG, electromyography; EOT, end of treatment; HbA1c, hemoglobin A1c; IQR, interquartile range; LDH, lactate dehydrogenase; mCCI, modified Charlson Comorbidity Index; MSI, microsatellite instability; MSI‐H, microsatellite instability‐high; MSS, microsatellite stable; PD, progressive disease.

^a^ Paclitaxel discontinuation indicates permanent cessation of paclitaxel after the fourth cycle due to neuropathy.

**FIGURE 1:**
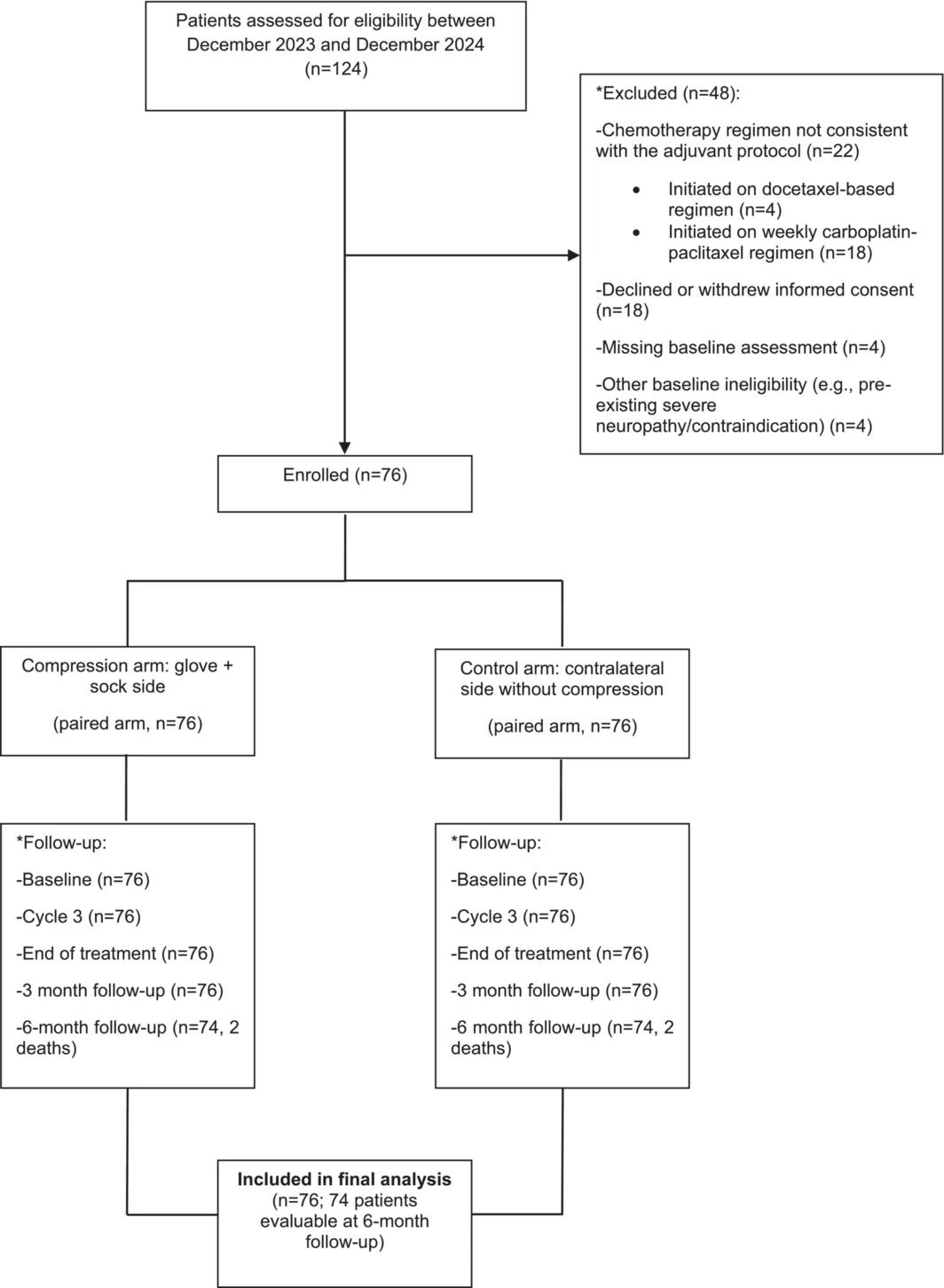
Study flow diagram of participants through enrollment, treatment, and follow‐up. A total of 124 patients were assessed for eligibility between December 2023 and December 2024; 76 were enrolled and completed paired‐arm assessments. Reasons for exclusion included nonstandard chemotherapy regimen, decline or withdrawal of informed consent, missing baseline assessments, and other ineligibilities. Follow‐up assessments were completed at baseline, Cycle 3, end of treatment, 3‐month, and 6‐month follow‐up. At the 6‐month assessment, data were available for 74 patients; assessments were not available for two patients due to death.

During the study period, disease progression occurred in two patients (2.6%) while on treatment. Among the patients who completed chemotherapy without progression, disease recurrence was observed in 14 patients (18.4%) during the posttreatment follow‐up phase. Death occurred in two patients (2.6%). Median DFS and OS were not reached. The 12‐month DFS rate was 88.2% (95% CI, 79.9%–96.5%; 7 events) and the 12‐month OS rate was 96.1% (95% CI, 90.7%–100.0%; 2 deaths). The study flow diagram and timing of assessments are shown in Figure 1.

### 3.2 Effect of Mechanical Compression on CIPN

**FIGURE 2:**
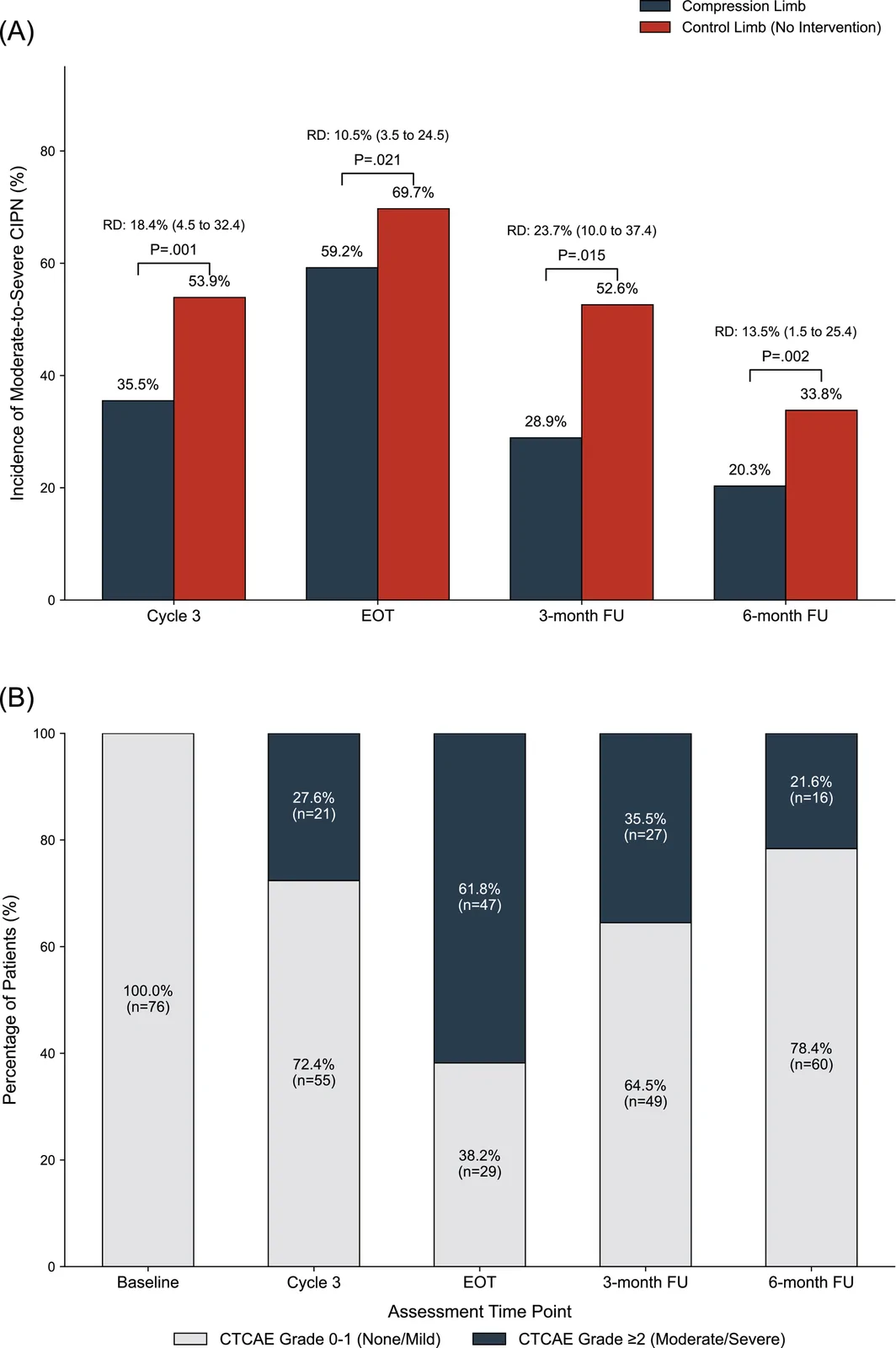
Effect of mechanical cCompression on limb‐specific chemotherapy‐induced peripheral neuropathy (CIPN). ANOVA, analysis of variance; CI, confidence interval; CIPN, chemotherapy‐induced peripheral neuropathy; EORTC QLQ‐CIPN20, European Organisation for Research and Treatment of Cancer Quality of Life Questionnaire–CIPN 20. (A) displays hand sensory CIPN scores over time in the mechanical compression and control arms. (B) displays foot sensory CIPN scores over time in the mechanical compression and control arms. Solid lines represent the compression arm, and dashed lines represent the control arm. Between‐group differences over time were evaluated using repeated‐measures analysis of variance with Greenhouse–Geisser correction.

No patients had CTCAE v5.0–defined moderate‐to‐severe (grade ≥ 2) CIPN at baseline in either limb (Figure 2, Panel A). During treatment, at Cycle 3, moderate‐to‐severe CIPN occurred in 27 of 76 compression limbs (35.5%) and 41 of 76 control limbs (53.9%) (RD, 18.4% [95% CI, 4.5% to 32.4%]; *p* = 0.001). At EOT, rates increased to 45 of 76 (59.2%) in compression limbs and 53 of 76 (69.7%) in control limbs (RD, 10.5% [95% CI, 3.5% to 24.5%]; *p* = 0.021). After completion of chemotherapy, moderate‐to‐severe CIPN remained less frequent in compression limbs than in control limbs at both the 3‐month follow‐up (22 of 76 [28.9%] vs. 40 of 76 [52.6%]; RD, 23.7% [95% CI, 10.0% to 37.4%]; *p* = 0.015) and the 6‐month follow‐up (15 of 74 [20.3%] vs. 25 of 74 [33.8%]; RD, 13.5% [95% CI, 1.5% to 25.4%]; *p* = 0.002). Consistent with these limb‐level findings, clinician‐graded neuropathy based on CTCAE v5.0 demonstrated a significant increase from baseline across all subsequent assessments, including Cycle 3, EOT, and posttreatment follow‐up at 3 and 6 months (all *p* < 0.001 vs. baseline) (Figure 2, Panel B).

The number needed to treat to prevent one case of moderate‐to‐severe CIPN was five at Cycle 3, 10 at EOT, four at 3‐month follow‐up, and seven at 6‐month follow‐up. Between‐limb differences in EORTC QLQ‐CIPN20 sensory scores approached or exceeded the established minimal clinically important difference of 2.5–5.9 points at EOT and 6‐month follow‐up time points. The absolute between‐group difference at EOT (~10%) should be interpreted cautiously, as it reflects a direct within‐patient comparison in which systemic confounders are inherently controlled.

### 3.3 Limb‐Specific Assessment of Sensory Neuropathy

**FIGURE 3:**
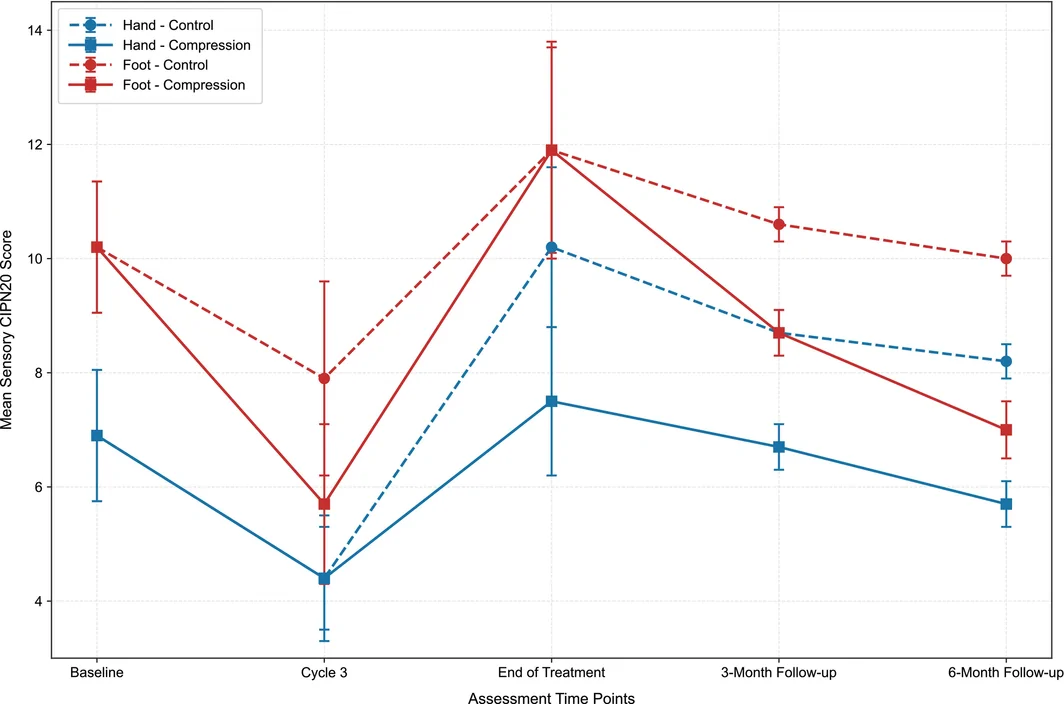
Limb‐specific trajectory of patient‐reported sensory neuropathy over time. CI, confidence interval; CIPN, chemotherapy‐induced peripheral neuropathy; EORTC QLQ‐CIPN20, European Organization for Research and Treatment of Cancer Quality of Life Questionnaire–Chemotherapy‐Induced Peripheral Neuropathy 20; EOT, end of treatment. Data are presented as mean (SD) scores derived from the sensory subscale of the EORTC QLQ‐CIPN20 (range, 0–100), with higher scores indicating worse neuropathy. The consistent gap between the red (foot) and blue (hand) trajectories illustrates the length‐dependent nature of taxane‐induced neuropathy, with lower extremities being more severely affected than upper extremities regardless of intervention (*p* < 0.001 at all post‐baseline time points). The separation between the solid (compression) and dashed (control) lines demonstrates the protective effect of the intervention, which was statistically significant for the hand at EOT and follow‐up, and for the foot during the follow‐up period.

To evaluate the efficacy of mechanical compression at distinct anatomical sites, we analyzed sensory neuropathy scores separately for upper and lower extremities (Figure 3 and Table S1). In the upper extremities, mechanical compression was associated with significantly lower mean sensory CIPN20 scores compared with the control limb starting from the EOT (mean, 7.5 vs. 10.2; *p* < 0.001) and persisting through the 3‐month (6.7 vs. 8.6; *p* < 0.001) and 6‐month follow‐up periods (5.7 vs. 8.2; p < 0.001).

In the lower extremities, no significant difference was observed at EOT (mean, 11.9 vs. 12.0; *p* = 0.835). However, mean sensory scores were significantly lower in the compression limb at 3‐month (8.7 vs. 10.7; *p* < 0.001) and 6‐month follow‐up (7.0 vs. 10.0; *p* < 0.001).

Analysis of limb‐specific differences showed that sensory scores were significantly higher in the lower extremities compared with the upper extremities across all time points, regardless of trial arm (Figure 3). Specifically, at the EOT, mean sensory scores in the feet were significantly higher than in the hands for both the control (12.0 vs. 10.2; *p* < 0.001) and compression (11.9 vs. 7.5; *p* < 0.001) sides (Table S1).

### 3.4 Longitudinal Changes in CIPN Severity Based on EORTC QLQ‐CIPN20 Scores

**FIGURE 4:**
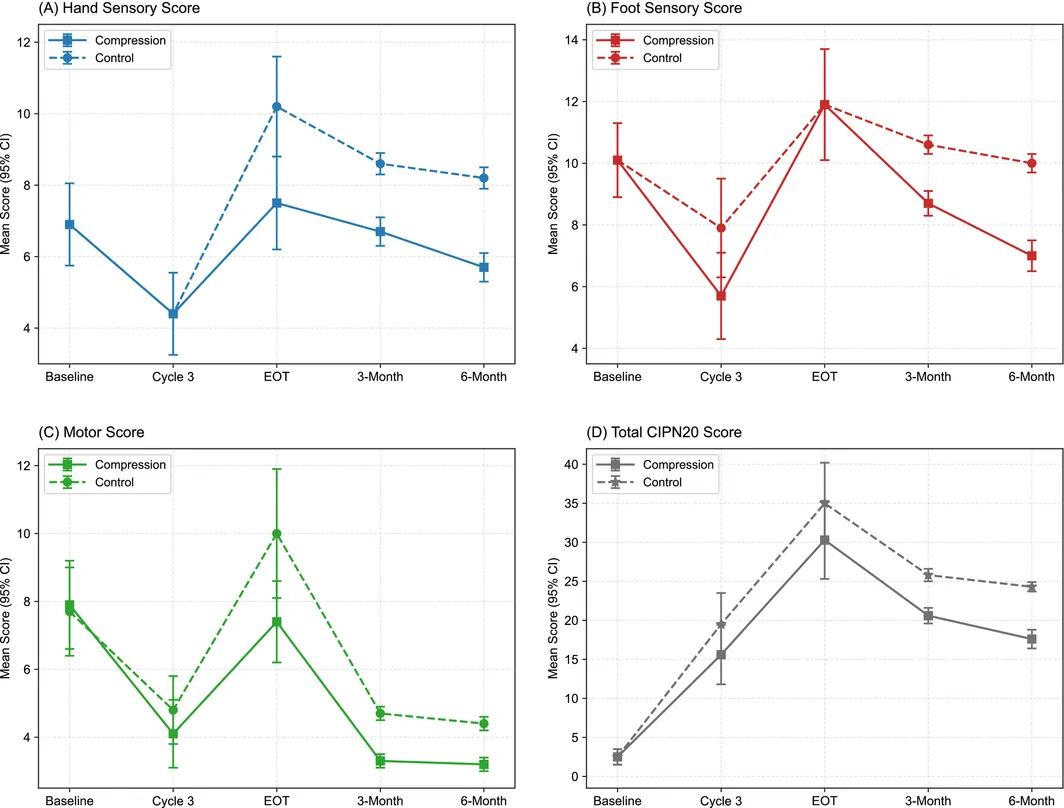
Longitudinal trajectories of EORTC QLQ‐CIPN20 Scores by domain. ANOVA, analysis of variance; CI, confidence interval; CIPN, chemotherapy‐induced peripheral neuropathy; EMM, estimated marginal mean; EORTC QLQ‐CIPN20, European Organization for Research and Treatment of Cancer Quality of Life Questionnaire–Chemotherapy‐Induced Peripheral Neuropathy 20; EOT, end of treatment. Estimated marginal means (EMMs) with 95% confidence intervals are shown for (A) hand sensory scores, (B) foot sensory scores, (C) motor scores, and (D) total scores across 5 time points: Baseline, Cycle 3, end of treatment (EOT), 3‐month follow‐up, and 6‐month follow‐up. Solid lines represent the compression arm; dashed lines represent the control arm. (A) In the hand sensory domain, separation between arms begins at EOT (P for interaction = 0.003). (B) In the foot sensory domain, separation is delayed, emerging during follow‐up (*p* for interaction = 0.018). (C) Motor scores show a significant time‐by‐arm interaction (*p* = 0.041). (D) Total scores reflect the cumulative burden (*p* < 0.001). Repeated‐measures ANOVA with Greenhouse–Geisser correction was used for analysis.

Longitudinal changes in patient‐reported neuropathy severity assessed by the EORTC QLQ‐CIPN20 are shown in Figure 4, with repeated‐measures analysis of variance results summarized in Table S2. Total EORTC QLQ‐CIPN20 scores increased over time in both compression and control limbs, remaining consistently lower in compression limbs at all post‐baseline assessments (Figure 4D).

Time‐by‐limb interactions were observed for hand sensory (*p* = 0.003), foot sensory (*p* = 0.018), and motor scores (*p* = 0.041) (Table S2). Sensory and motor CIPN20 scores increased during treatment and partially improved during follow‐up, with lower scores in compression limbs across post‐baseline time points (Figure 4A–C).

Between‐limb effect sizes and within‐patient agreement across time points are reported in Table S3. Transitions in neuropathy severity categories based on CTCAE grading and EORTC QLQ‐CIPN20 thresholds are presented in Figure 2 and Table S4.

### 3.5 Agreement Between Clinician‐Graded and Patient‐Reported Neuropathy

Spearman correlations between CTCAE grade (≥ 2) and EORTC QLQ‐CIPN20 scores are summarized in Table S5. At EOT, correlations in the compression limb were *ρ* = 0.860 for total and motor scores (both *p* < 0.001). Agreement between clinician‐graded neuropathy (CTCAE grade ≥ 2) and patient‐reported neuropathy severity (EORTC QLQ‐CIPN20 total score ≥ 25) across treatment and follow‐up time points is summarized in Table S6.

ROC analyzes for EORTC QLQ‐CIPN20 domains against CTCAE grade ≥ 2 are shown in Figure S3 and Table S7. For sensory scores at Cycle 3, the AUC was 0.922 (95% CI, 0.861–0.983) in the compression limb and 0.906 (95% CI, 0.835–0.976) in the control limb; at EOT, the AUC was 0.965 (95% CI, 0.928–1.000) and 0.977 (95% CI, 0.952–1.000), respectively.

### 3.6 Electrophysiologic Correlation With Clinical and Patient‐Reported Neuropathy

At the EOT, electrophysiologic evaluation was performed in a subset of patients to assess concordance between EMG findings and clinical and patient‐reported neuropathy measures. EMG‐confirmed peripheral neuropathy was present in 12 of 32 patients (37.5%) with CTCAE v5.0 grade ≥ 2 neuropathy, compared with 2 of 15 patients (13.3%) with grade 0–1 (*p* = 0.015; OR, 3.90; 95% CI, 1.75–8.70). Similarly, EMG‐confirmed neuropathy was detected in 11 of 31 patients (35.5%) with an EORTC QLQ–CIPN20 total score ≥ 25 and in three of 16 patients (18.8%) with a score < 25 (*p* = 0.009; OR, 2.38; 95% CI, 1.30–4.35).

Agreement between EMG findings and CIPN20 classification was moderate to good (*κ* = 0.62; *p* < 0.001). EMG‐confirmed neuropathy correlated with both CTCAE grading (Spearman *ρ* = 0.52; *p* = 0.012) and total CIPN20 scores (*ρ* = 0.42; *p* = 0.031).

### 3.7 Factors Associated With Moderate‐to‐Severe CIPN

**FIGURE 5:**
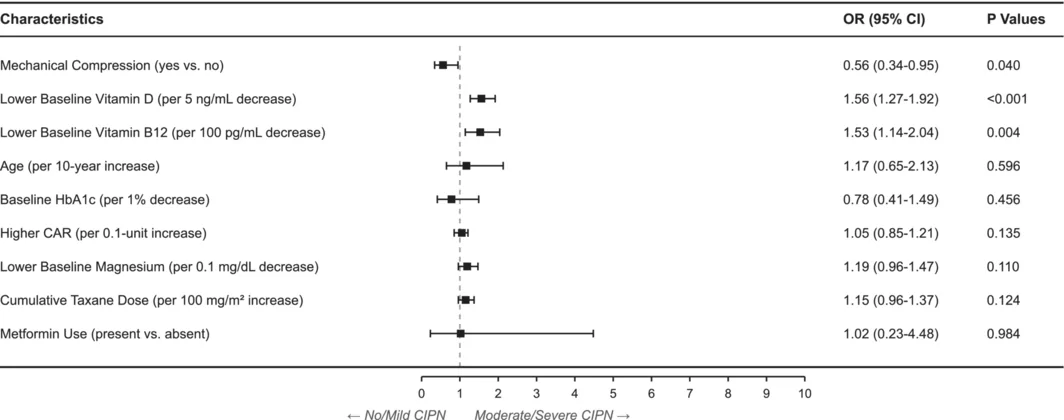
Multivariable GEE analysis of factors associated with moderate‐to‐severe chemotherapy‐induced peripheral neuropathy (CIPN) at end of treatment (EOT). CIPN, chemotherapy‐induced peripheral neuropathy; GEE, generalized estimating equation; OR, odds ratio; CI, confidence interval; CRP, C‐reactive protein; CAR, C‐reactive protein–to–albumin ratio. Forest plot showing adjusted odds ratios (ORs) and 95% confidence intervals from a multivariable generalized estimating equation (GEE) model evaluating factors associated with moderate‐to‐severe CIPN at the EOT. The model accounted for within‐patient correlation by clustering on patient ID and used an exchangeable working correlation structure. Odds ratios greater than 1 indicate higher odds of CIPN, whereas odds ratios less than 1 indicate lower odds of CIPN. For selected biomarkers (vitamin D, vitamin B12, and magnesium), odds ratios are expressed per unit decrease in baseline levels to facilitate clinical interpretation. *Variance inflation factors (VIFs) and tolerance values were examined to assess multicollinearity. All variables had VIF values < 5 and tolerance values > 0.30, indicating no evidence of significant multicollinearity.

Univariable associations with moderate‐to‐severe CIPN at EOT are reported in Table S8. Increasing age (per 10 years: OR, 1.71 [95% CI, 1.20–2.44]; *p* = 0.003), higher HbA1c (per 1%: OR, 1.77 [95% CI, 1.18–2.65]; *p* = 0.006), lower magnesium (per 0.1 mg/dL: OR, 0.90 [95% CI, 0.81–0.99]; *p* = 0.031), lower vitamin B12 (per 100 pg/mL: OR, 0.72 [95% CI, 0.58–0.88]; *p* = 0.003), and lower vitamin D levels (per 5 ng/mL: OR, 0.63 [95% CI, 0.51–0.77]; *p* < 0.001) were associated with CIPN EOT. In multivariable generalized estimating equation models, mechanical compression was associated with lower odds of moderate‐to‐severe CIPN at EOT (adjusted OR, 0.56; 95% CI, 0.34–0.95; *p* = 0.040). Lower baseline vitamin D (per 5 ng/mL decrease; adjusted OR, 1.56; 95% CI, 1.27–1.92; *p* < 0.001) and vitamin B12 levels (per 100 pg/mL decrease; adjusted OR, 1.53; 95% CI, 1.14–2.04; *p* = 0.004) were independently associated with higher odds of CIPN (Figure 5). The exploratory RF analysis corroborated the multivariable model, identifying baseline vitamin levels and the use of mechanical compression as the most influential predictors of CIPN severity. Exploratory RF model results are shown in Figures S4 and S5.

## 4 Discussion

This first prospective, self‐controlled study provides evidence on the prevention and characterization of CIPN in women receiving paclitaxel‐based chemotherapy for gynecologic malignancies. Unlike prior investigations largely limited to hand‐only cooling or compression, this study evaluated a dual‐limb mechanical compression strategy involving the nondominant hand and ipsilateral lower extremity, enabling assessment of both upper‐ and lower‐extremity neuropathy. Precisely reflecting this dual‐site approach, our results demonstrated distinct protective profiles for the upper and lower extremities. Mechanical compression provided immediate and sustained protection in the hands, significantly reducing neuropathy burden starting from the EOT. In the feet, the site most vulnerable to length‐dependent taxane toxicity, although the protective benefit was not evident at the immediate EOT, a significant clinical benefit emerged during the recovery phase at 3‐ and 6‐month follow‐ups. These findings indicate a sustained reduction in posttreatment neuropathy burden across both limb sets. In addition, EORTC QLQ‐CIPN20 demonstrated good discriminative performance for identifying CTCAE‐defined neuropathy across treatment and follow‐up time points, supporting the utility of patient‐reported outcomes for longitudinal CIPN assessment.

Our findings extend the heterogeneous literature on non‐pharmacologic prevention of taxane‐induced peripheral neuropathy. Existing evidence for compression or cooling interventions derives predominantly from breast cancer populations, despite differences in treatment context, surgical burden, and baseline risk of lower‐extremity neuropathy in gynecologic oncology [14, 16, 18, 23]. The POLAR randomized clinical trial demonstrated that both surgical glove–based compression and cryotherapy reduce taxane‐related neuropathy [13]. Although both approaches are clinically feasible, cooling typically requires dedicated equipment, whereas compression can be delivered using readily available materials, making it more scalable. A recent randomized clinical trial demonstrated that continuous foot cooling during paclitaxel infusion significantly reduced CIPN incidence and severity in patients with advanced breast cancer, with benefit sustained through follow‐up [24]. These findings support the biological rationale for localized perfusion‐based neuroprotection in lower extremities and complement the present results. Results for compression therapy, however, have been inconsistent, with some studies reporting reductions in Grade 2 or higher sensory neuropathy [14, 21, 23] and others, including a double‐blind phase II trial, showing no significant preventive effect [25]. These discrepancies likely reflect heterogeneity in study design, patient populations, and compression protocols. Notably, most prior studies focused exclusively on upper extremities, despite the substantial burden of lower‐extremity neuropathy in taxane‐treated patients [17]. A randomized clinical trial showing that neuromuscular exercise training during chemotherapy lowers CIPN risk further supports the hypothesis that interventions influencing peripheral perfusion, metabolic stress, and axonal recovery can modify neuropathy development [26]. Our results extend this concept by suggesting that mechanical compression may confer limb‐specific neuroprotection, particularly in anatomically vulnerable distal nerves. Moreover, our population consisted predominantly of patients undergoing major abdominal or pelvic surgery, a context in which perioperative factors may further increase peripheral nerve vulnerability [15, 27, 28, 29]. By applying simultaneous compression to both upper and lower limbs, this study specifically addresses these critical gaps. Our results suggest that mechanical compression may provide protection not only in the hands but also in the lower extremities, a site historically prone to severe, length‐dependent toxicity and surgical stress, particularly during the posttreatment recovery phase.

The association between mechanical compression and reduced neuropathy should be interpreted in the context of the complex pathophysiology of CIPN. Axonal degeneration, mitochondrial dysfunction, microtubule disruption, and preferential involvement of small sensory fibers contribute to symptom development and are not fully explained by reduced local drug exposure alone [6, 11, 30]. Proposed mechanisms of compression‐based interventions include transient vasoconstriction, modulation of capillary permeability, and attenuation of neurogenic inflammation [15]. Although paclitaxel acts systemically, the neuroprotective mechanism of compression is locally mediated through transient reduction of limb microvascular perfusion, which supports the biological plausibility of unilateral protection. Potential crossover effects on the contralateral limb cannot be entirely excluded; however, such effects would be expected to attenuate rather than amplify the observed between‐limb differences, suggesting that the current findings are conservative rather than inflated. In a clinically selected subset, electrophysiological assessments showed concordance with clinical severity but did not demonstrate significant differences between compressed and control extremities, consistent with the limited sensitivity of standard nerve conduction studies for detecting small‐fiber dysfunction [28]. The persistence of patient‐reported sensory symptoms despite limited electrophysiological differences underscores the complementary value of patient‐reported outcome measures [19, 30]. The delayed emergence of benefit in the lower extremities may reflect the length‐dependent nature of axonal recovery, whereby distal nerves require prolonged periods for regeneration, although measurement variability and natural symptom resolution cannot be excluded as contributing factors. Approximately one‐fifth of patients continued to experience moderate‐to‐severe neuropathy at 6 months, consistent with the known long‐term burden of CIPN [9, 10, 11]. The sustained separation between compression and control extremities during follow‐up, particularly in the lower limbs, highlights the importance of longitudinal, symptom‐focused monitoring given the association of lower‐extremity neuropathy with gait impairment and functional limitation [31].

Beyond the mechanical intervention, this study highlights the potential contribution of baseline clinical and laboratory variables to identify patients at increased risk of CIPN. Lower baseline vitamin D and vitamin B12 levels were independently associated with the development of moderate‐to‐severe neuropathy. These findings align with prior observational and translational studies linking nutritional and metabolic vulnerability to CIPN [8, 29, 32, 33, 34]. Previous large patient series have similarly reported associations between micronutrient imbalance and paclitaxel‐related neuropathy severity, suggesting that these deficiencies may represent modifiable risk factors worthy of further investigation, although residual confounding cannot be excluded and results have varied across populations [7, 8, 21, 29, 32, 35, 36]. In exploratory RF analyzes, vitamin D, vitamin B12, cumulative taxane dose, and BMI emerged among the most influential predictors; these findings should be considered hypothesis‐generating and require external validation. The alignment of machine learning‐based (ML) importance rankings with multivariable regression results reinforces the robustness of these biological associations. Furthermore, the ability of the RF model to incorporate variables like BMI and cumulative dose likely reflects the strength of data‐driven approaches in capturing nonlinear associations and correlated risk patterns, supporting a complementary role for ML alongside traditional regression methods in CIPN risk assessment [30, 37, 38].

This study has several strengths, including its prospective design, focus on an underrepresented gynecologic oncology population, and the innovative self‐controlled dual‐limb compression approach that enabled within‐patient comparisons, thereby minimizing systemic confounding. The incorporation of both clinician‐graded and patient‐reported neuropathy outcomes, along with standardized follow‐up extending to 6 months, allowed for the comprehensive evaluation of both acute and persistent CIPN phenotypes. Limitations include the inability to blind the visible intervention, which may introduce detection bias, particularly for subjective patient‐reported outcomes. The strong concordance between clinician‐graded CTCAE scores and EORTC QLQ‐CIPN20 scores across all time points supports the internal consistency of the findings and argues against a systematic reporting bias. Compression was consistently applied to the nondominant limb rather than through randomized limb allocation, which introduces the possibility that functional asymmetry between dominant and nondominant extremities may have influenced neuropathy perception or reporting. Although prior evidence suggests limb dominance has minimal effect on taxane‐induced neuropathy severity, this cannot be fully excluded as a source of confounding, and future studies should consider randomized limb assignment [39]. Other limitations include the single‐center design, the exclusion of patients with preexisting neuropathy, which limits generalizability to treatment‐naive populations, the restriction of electrophysiological assessment to a clinically selected subset of symptomatic patients who were referred by the consulting neurologist and consented to the procedure, which may have introduced verification bias and prevented detection of subclinical neuropathy, and the exploratory nature of the predictive modeling, which limits causal inference and requires external validation in larger cohorts.

## 5 Conclusion

In this prospective self‐controlled study, dual‐site mechanical compression of the nondominant hand and ipsilateral lower‐extremity was associated with a lower burden of sensory CIPN in women receiving paclitaxel‐based chemotherapy. The protective benefit exhibited a limb‐specific pattern: immediate and sustained in the upper extremities and emerging during the posttreatment recovery phase in the lower extremities. Patient‐reported neuropathy measures showed concordance with clinician grading, supporting their use for longitudinal CIPN assessment. In exploratory analyzes, routinely available metabolic and treatment‐related variables demonstrated discriminatory ability for identifying patients at higher risk. These findings suggest that dual‐limb compression may represent a feasible, low‐cost, and scalable preventive approach for CIPN. Larger, multicenter randomized trials are needed to confirm these findings.

## Author Contributions

**Kadriye Başkurt:** conceptualization, investigation, writing – original draft, writing – review and editing, project administration, data curation, resources. **Galip Can Uyar:** formal analysis, software, methodology. **Enes Yeşilbaş:** methodology, data curation. **Fatma Zehra Altunç:** data curation. **Damla Erimhan Çevik:** data curation. **İsmet Murat Melek:** data curation. **Yasemin Eren:** data curation. **Ömür Berna Çakmak Öksüzoğlu:** supervision. **Kadriye Bir Yücel:** supervision. **Antonio Di Meglio:** writing – review and editing. **Isabelle Ray‐Coquard:** writing – review and editing. **Osman Sütcüoğlu:** supervision, writing – review and editing, conceptualization, methodology, project administration.

## Ethics Statement

The study was approved by the Etlik City Hospital Ethics Committee (AEŞH‐EK1‐2023‐828), conducted in accordance with the Declaration of Helsinki, and prospectively registered at ClinicalTrials.gov (NCT07105553). Written informed consent was obtained from all participants.

## Conflicts of Interest

Dr. Isabelle Ray‐Coquard reported grants/contracts from AstraZeneca, Clovis, GlaxoSmithKline (GSK), Mersana, Bristol Myers Squibb (BMS), and Merck Sharp & Dohme (MSD); honoraria/speakers bureaus from AstraZeneca, Clovis, GSK, Mersana, BMS, MSD, Roche, PharmaMar, Seagen, Eisai, Novartis, DSI, AbbVie, Lilly, Tubulis, Genmab, and TORL123; travel support from Roche, GSK, AstraZeneca, PharmaMar, and MSD; and advisory board participation with Clovis, Deciphera, Adaptimmune, Sutro, ImmunoGen, AbbVie, DSI, Tubulis, AstraZeneca, GSK, MSD, Lilly, Scorpion, and Antares, all outside the submitted work, as detailed in the ICMJE disclosure form. The other authors declare no conflicts of interest.

## Supporting information


**Table S1:** Limb‐Specific Mean Sensory Neuropathy Scores and statistical comparisons over time.
**Table S2:** Repeated‐measures ANOVA results for EORTC QLQ‐CIPN20 Domain Scores.
**Table S3:** Between‐limb effect sizes and within‐patient agreement across time points.
**Table S4:** Transitions in neuropathy severity categories based on CTCAE grading and EORTC QLQ‐CIPN20 thresholds.
**Table S5:** Spearman correlations between CTCAE Grade and EORTC QLQ‐CIPN20 Scores.
**Table S6:** Agreement between clinician‐graded and patient‐reported neuropathy.
**Table S7:** ROC analysis results for EORTC QLQ‐CIPN20 domains against CTCAE grade ≥ 2.
**Table S8:** Univariable associations with moderate‐to‐severe CIPN at end of treatment.
**Figure S1:** Measurement methods for hand, ankle, and calf circumference with corresponding sizing and compression classifications.
**Figure S2:** Longitudinal transitions of neuropathy severity across time points based on CTCAE and CIPN20 Scores.
**Figure S3:** Receiver operating characteristic curves for EORTC QLQ‐CIPN20 Sensory, Motor, and Total Scores against CTCAE v5.0 neuropathy grading.
**Figure S4:** Variable importance derived from random forest models predicting moderate‐to‐severe CIPN at end of treatment.
**Figure S5:** Receiver operating characteristic curves for random forest models predicting moderate‐to‐severe CIPN at end of treatment.
Appendix 1. TREND Statement Checklist.

## Data Availability

The data that support the findings of this study are available from the corresponding author upon reasonable request.
