# Supplementary material for: Limb Compression Therapy and Chemotherapy‐Induced Peripheral Neuropathy in Women With Gynecologic Cancers: A Prospective Self‐Controlled Study(NEURO‐GLOVE Trial)
Source: Int J Cancer. 2026 Jun 18;159(9):2300–13. doi: 10.1002/ijc.70601 (PMC13547992; doi:10.1002/ijc.70601)
Supplement: Supplementary file 1 [file IJC-159-2300-s001.pdf]

Limb Compression Therapy and Chemotherapy-Induced Peripheral Neuropathy in Women with Gynecologic Cancers: A Prospective Self-Controlled Study (NEURO-GLOVE Trial)

Kadriye Başkurt, Galip Can Uyar, Enes Yeşilbaş, Fatma Zehra Altunç, Damla Erimhan Çevik, İsmet Murat Melek, Yasemin Eren, Ömür Berna Çakmak Öksüzoğlu, Kadriye Bir Yücel, Antonio Di Meglio, Isabelle Ray-Coquard, Osman Sütcüoğlu

Table of Contents

Supplementary Methods

Supplementary Table 1. Limb-Specific Mean Sensory Neuropathy Scores and Statistical Comparisons Over Time

Supplementary Table 2. Repeated-Measures ANOVA Results for EORTC QLQ-CIPN20 Domain Scores

Supplementary Table 3. Between-Limb Effect Sizes and Within-Patient Agreement Across Time Points

Supplementary Table 4. Transitions in Neuropathy Severity Categories Based on CTCAE Grading and EORTC QLQ-CIPN20 Thresholds

Supplementary Table 5. Spearman Correlations Between CTCAE Grade and EORTC QLQ-CIPN20 Scores

Supplementary Table 6. Agreement Between Clinician-Graded and Patient-Reported Neuropathy

Supplementary Table 7. ROC Analysis Results for EORTC QLQ-CIPN20 Domains Against CTCAE Grade ≥2

Supplementary Table 8. Univariable Associations with Moderate-to-Severe CIPN at End of Treatment

Supplementary Figure 1. Measurement Methods for Hand, Ankle, and Calf Circumference With Corresponding Sizing and Compression Classifications

Supplementary Figure 2. Longitudinal Transitions of Neuropathy Severity Across Time Points Based on CTCAE and CIPN20 Scores

Supplementary Figure 3. Receiver Operating Characteristic Curves for EORTC QLQ-CIPN20 Sensory, Motor, and Total Scores Against CTCAE v5.0 Neuropathy Grading

Supplementary Figure 4. Variable Importance Derived From Random Forest Models Predicting Moderate-to-Severe CIPN at End of Treatment

Supplementary Figure 5. Receiver Operating Characteristic Curves for Random Forest Models Predicting Moderate-to-Severe CIPN at End of Treatment

Appendix 1. TREND Statement Checklist

Supplementary Methods

CIPN20 Scoring, Subscale Computation, and Definition of Neuropathy Severity

EORTC QLQ-CIPN20 Instrument

Chemotherapy-induced peripheral neuropathy was assessed using the European Organisation for Research and Treatment of Cancer Quality of Life Questionnaire–Chemotherapy-Induced Peripheral Neuropathy 20-item scale (EORTC QLQ-CIPN20). The instrument consists of:

9 sensory items, 8 motor items, and 3 autonomic items,

each rated on a 4-point Likert scale (1 = not at all to 4 = very much). All 20 items were administered at every study time point.

Autonomic items, including the two sex-specific genital items, were not included in statistical analyses, because autonomic neuropathy was not a prespecified endpoint, autonomic symptoms were infrequent, and genital items are not uniformly applicable across participants. Per EORTC scoring guidance, exclusion of non-applicable autonomic items does not compromise the validity of sensory, motor, hand, foot, or total CIPN20 scores.

Scoring Procedure

For each domain, the raw mean score (RS) was calculated as the average of the relevant item responses. All domains were linearly transformed to a 0–100 EORTC standard scale:

Higher scores indicate more severe neuropathy. A total CIPN20 score was computed as the mean of all 20 items and transformed using the same formula.

Definition of Clinically Significant CIPN

Based on prior literature, moderate-to-severe CIPN was defined as a total CIPN20 score ≥25. This threshold was applied consistently across all timepoints (baseline, post-cycle 3, end of treatment, and 6-month follow-up).

Sensitivity analyses using alternative thresholds (≥20 and ≥30) showed similar effect directions and were not used as primary endpoints.

Hand (Upper-Extremity) and Foot (Lower-Extremity) Anatomical Subscores

To investigate regional effects of compression therapy, anatomically localized subscores were derived from CIPN20, using item numbers verified from the original questionnaire.

Hand (Upper-Extremity) Subscore — 7 items

- 31 – Tingling in fingers or hands
- 33 – Numbness in fingers or hands
- 35 – Shooting or burning pain in fingers or hands
- 37 – Hand cramps
- 41 – Difficulty holding a pen
- 42 – Difficulty manipulating small objects
- 43 – Difficulty opening jars or bottles

Foot (Lower-Extremity) Subscore — 7 items

- 32 – Tingling in toes or feet
- 34 – Numbness in toes or feet
- 36 – Shooting or burning pain in toes or feet
- 38 – Foot cramps
- 39 – Difficulty standing or walking due to sensory loss
- 44 – Foot drop
- 45 – Difficulty climbing stairs or rising from a chair

For both hand and foot domains, subscale scores were computed by averaging the included items and transforming to a 0–100 scale. Higher scores reflect more severe regional neuropathy.

Autonomic Items

Autonomic items (40, 46, 47) were recorded but excluded from primary analyses, as autonomic neuropathy was not a study endpoint and symptoms were infrequent. Item 50 (erectile dysfunction) was not analyzed because it applies only to male patients and is not relevant in this predominantly female cohort.

Supplementary Statistical Methods

Use of EORTC QLQ-CIPN20 Scores in Analyses

EORTC QLQ-CIPN20 sensory, motor, hand, foot, and total scores were analyzed as both continuous variables and dichotomized outcomes. Dichotomization was based on a predefined threshold for clinically significant neuropathy (total CIPN20 score ≥25). For analyses evaluating regional effects of mechanical compression, only anatomically localized hand (upper-extremity) and foot (lower-extremity) subscores were used, as these corresponded directly to the limbs receiving compression.

Agreement Between Clinician-Graded and Patient-Reported Neuropathy

Agreement between clinician-assessed neuropathy severity (CTCAE grade ≥2 vs <2) and patient-reported neuropathy severity (EORTC QLQ-CIPN20 total score ≥25 vs <25) was evaluated using Cohen's κ statistic, percent agreement, and prevalence-adjusted bias-adjusted κ (PABAK). Associations between CTCAE grades and continuous EORTC QLQ-CIPN20 domain scores were examined using Spearman rank correlation coefficients.

Discriminative Performance Analyses

The ability of EORTC QLQ-CIPN20 sensory, motor, hand, foot, and total scores to discriminate clinically significant neuropathy (CTCAE grade ≥2) was assessed using receiver operating characteristic (ROC) curve analyses. Area under the curve (AUC) estimates with 95% confidence intervals were calculated.

Longitudinal Trend and Effect Size Analyses

Transitions in CTCAE neuropathy grades across time were evaluated using chi-square tests for trend, with Cramér's V reported as a measure of association strength. Between-limb effect sizes were summarized using Cohen's d, and within-patient agreement across repeated measurements was assessed using intraclass correlation coefficients (ICCs). For repeated-measures analyses, Greenhouse–Geisser corrections were applied when sphericity assumptions were violated, and partial eta-squared values were reported.

Electrophysiologic Correlation Analyses

In patients who underwent electrophysiologic evaluation, agreement between electromyography (EMG)–confirmed peripheral neuropathy and clinical neuropathy classifications was assessed using Cohen's κ statistic. Associations between EMG findings and continuous clinical measures, including CTCAE neuropathy grades and total EORTC QLQ-CIPN20 scores, were examined using Spearman rank correlation coefficients.

Exploratory Machine Learning Analyses

As an exploratory, hypothesis-generating analysis, random forest models were constructed to rank the relative importance of baseline clinical and laboratory variables associated with moderate-to-severe neuropathy. Variable importance was quantified using mean decrease in Gini impurity. These analyses were not intended for individual-level prediction.

Supplementary Table 1. Limb-Specific Mean Sensory Neuropathy Scores and Statistical Comparisons Over Time

Time Point	HAND Compression Mean (SD)	HAND Control Mean (SD)	P Value (Comp vs Cont)	FOOT Compression Mean (SD)	FOOT Control Mean (SD)	P Value (Comp vs Cont)	Hand vs Foot P Value (Comp Side)	Hand vs Foot P Value (Control Side)
**Cycle 3**	4.4 (4.9)	4.6 (4.4)	.511	5.7 (6.2)	7.9 (7.4)	**.005**	**<.001**	**<.001**
**EOT**	7.5 (6.0)	10.2 (6.2)	**<.001**	11.9 (8.7)	12.0 (8.0)	.835	**<.001**	**<.001**
**3-Month Follow-up**	6.7 (7.2)	8.6 (8.5)	**<.001**	8.7 (7.6)	10.7 (8.3)	**<.001**	**<.001**	**<.001**
**6-Month Follow-up**	5.7 (6.6)	8.2 (8.4)	**<.001**	7.0 (7.1)	10.0 (8.7)	**<.001**	**<.001**	**<.001**

Abbreviations: CIPN, chemotherapy-induced peripheral neuropathy; Comp, compression; Cont, control; EORTC QLQ-CIPN20, European Organisation for Research and Treatment of Cancer Quality of Life Questionnaire–Chemotherapy-Induced Peripheral Neuropathy 20; EOT, end of treatment; SD, standard deviation

Data are presented as mean (SD) scores derived from the sensory subscale of the EORTC QLQ-CIPN20 (range, 0-100), with higher scores indicating worse neuropathy. [a] Calculated using the paired Wilcoxon signed-rank test. The "Comp vs Cont" columns compare the compression limb with the control limb at the same anatomical site (hand vs hand; foot vs foot). The "Hand vs Foot" columns compare the upper and lower extremities within the same study arm (compression or control) to assess length-dependent neuropathy patterns. Statistically significant p values (<.05) are indicated in bold.

Supplementary Table 2. Repeated measures ANOVA results for sensory, motor and total EORTC QLQ-CIPN20 scores

Outcome	Time point	Compression Mean (95% CI)	Control Mean (95% CI)	Comp vs Control (p)	Comp–Control Mean Difference (95% CI)	Overall ANOVA effects (F, p, η²)	Post-hoc within-arm changes (p, Bonferroni)
**Total CIPN**	**Baseline**	2.42 (1.44–3.41)	2.42 (1.44–3.41)	—	—	**Time:** F (1.93,140.5) =73.6, p<0.001, η²=0.502	Baseline → Cycle 3: p<0.001
	**Cycle 3**	14.69 (10.95–18.43)	19.80 (15.54–24.06)	0.062	−5.11 (−9.8 to −0.4)	**Arm:** F (1,73) =58.6, p<0.001, η²=0.445	Cycle 3 → EOT: p<0.001
	**End of treatment**	29.48 (24.33–34.62)	34.15 (28.96–39.33)	**0.001**	−4.68 (−7.2 to −2.1)	**Time×Arm:** F (2.18,158.7) =5.17, p=0.005, η²=0.066	Baseline → EOT: p<0.001
	**3-mo Follow-up**	20.60 (19.72–21.49)	25.78 (25.14–26.42)	**<0.001**	−5.18 (−6.02 to –4.33)		EOT → 3-mo: p<0.001
	**6-mo Follow-up**	17.71 (16.60–18.82)	24.23 (23.58–24.88)	**<0.001**	−6.52 (−7.8 to −5.2)		3-mo → 6-mo: Compression p<0.001; Control p<0.001
**Total Sensory CIPN**	**Baseline**	8.75 (7.47–10.02)	8.75 (7.47–10.02)	—	—	**Time:** F (2.34,170.5) =34.75, p<0.001, η²=0.322	Baseline → Cycle 3: p<0.001
	**Cycle 3**	9.52 (7.08–11.96)	12.50 (9.76–15.24)	**0.041**	−2.98 (−5.9 to −0.1)	**Arm:** F (1,73) =71.95, p<0.001, η²=0.496	Cycle 3 → EOT: p<0.001
	**End of treatment**	18.92 (15.58–22.25)	20.98 (18.18–23.79)	**0.018**	−2.06 (−3.8 to −0.4)	**Time×Arm:** F (2.23,162.4) =9.81, p<0.001, η²=0.118	Baseline → EOT: p<0.001
	**3-mo Follow-up**	15.53 (14.88–16.17)	19.38 (18.88–19.87)	**<0.001**	−3.85 (−4.7 to −3.0)		EOT → 3-mo: p<0.001
	**6-mo Follow-up**	12.52 (11.73–13.31)	18.24 (17.76–18.71)	**<0.001**	−5.72 (−6.8 to −4.7)		3-mo → 6-mo: Compression p<0.001; Control p<0.001
**Hand Sensory CIPN**	**Baseline**	7.00 (5.81–8.20)	7.00 (5.81–8.20)	—	—	**Time:** F (2.85,207.8) =15.96, p<0.001, η²=0.179	Baseline → Cycle 3: p=0.001
	**Cycle 3**	4.13 (3.07–5.21)	4.57 (3.54–5.59)	0.480	−0.44 (−1.6 to 0.7)	**Arm:** F (1,73) =104.0, p<0.001, η²=0.588	Cycle 3 → EOT: p=0.087
	**End of treatment**	7.34 (5.99–8.72)	10.00 (8.59–11.41)	**<0.001**	−2.66 (−4.4 to −1.0)	**Time×Arm:** F (2.54,185.3) =16.80, p<0.001, η²=0.187	Baseline → EOT: p=0.161
	**3-mo Follow-up**	6.72 (6.37–7.07)	8.66 (8.39–8.92)	**<0.001**	−1.94 (−2.3 to −1.5)		EOT → 3-mo: p<0.001
	**6-mo Follow-up**	5.73 (5.34–6.12)	8.21 (7.91–8.51)	**<0.001**	−2.48 (−2.9 to −2.1)		3-mo → 6-mo: Compression p<0.001; Control p<0.001
**Foot Sensory CIPN**	**Baseline**	10.26 (9.06–11.47)	10.26 (9.06–11.47)	—	—	**Time:** F (2.79,203.9) =13.49, p<0.001, η²=0.156	Baseline → Cycle 3: p<0.001
	**Cycle 3**	5.39 (4.02–6.76)	7.91 (6.20–9.63)	**0.020**	−2.52 (−4.6 to −0.4)	**Arm:** F (1,73) =47.58, p<0.001, η²=0.395	Cycle 3 → EOT: p=0.256
	**End of treatment**	11.57 (9.59–13.56)	11.63 (9.89–13.38)	0.912	−0.06 (−2.1 to 2.0)	**Time×Arm:** F (2.23,163.1) =10.03, p<0.001, η²=0.121	Baseline → EOT: p=0.063
	**3-mo**	8.73 (8.38–9.07)	10.65 (10.35–	**<0.001**	−1.93 (−2.3 to −1.5)		EOT → 3-mo: p<0.001

Outcome	Time point	Compression Mean (95% CI)	Control Mean (95% CI)	Comp vs Control (p)	Comp–Control Mean Difference (95% CI)	Overall ANOVA effects (F, p, η^2)	Post-hoc within-arm changes (p, Bonferroni)
	**Follow-up**		10.96)				
	**6-mo Follow-up**	7.03 (6.51–7.56)	10.04 (9.75–10.33)	**<0.001**	−3.01 (−3.6 to −2.3)		3-mo→6-mo: Compression p<0.001; Control p<0.001
**Motor CIPN**	**Baseline**	7.83 (6.50–9.10)	7.83 (6.50–9.10)	—	—	**Time:** F (2.29,167.1) =23.67, p<0.001, η^2=0.245	Baseline→Cycle 3: p<0.001
	**Cycle 3**	3.88 (2.87–4.88)	4.73 (3.71–5.75)	0.210	–0.85 (–1.9 to 0.3)	**Arm:** F (1,73) =36.55, p<0.001, η^2=0.334	Cycle 3→EOT: p<0.001
	**End of treatment**	7.21 (6.03–8.39)	9.80 (7.88–11.71)	**<0.001**	–2.59 (–4.3 to –0.9)	**Time×Arm:** F (1.72,125.9) =6.95, p=0.002, η^2=0.087	Baseline→EOT: p<0.001
	**3-mo Follow-up**	3.32 (3.15–3.48)	4.70 (4.57–4.83)	**0.005**	–1.38 (–1.6 to –1.2)		EOT→3-mo: p<0.001
	**6-mo Follow-up**	3.24 (2.93–3.54)	4.36 (4.19–4.52)	**0.016**	–1.12 (–1.4 to –0.8)		3-mo→6-mo: Compression p<0.001; Control p<0.001

Abbreviations: ANOVA = analysis of variance; CI = confidence interval; CIPN = chemotherapy-induced peripheral neuropathy; EORTC QLQ-CIPN20 = European Organisation for Research and Treatment of Cancer Quality of Life Questionnaire–Chemotherapy-Induced Peripheral Neuropathy 20; EOT = end of treatment; η^2 = partial eta squared.

Compression arm refers to the limb receiving both glove and sock application; the contralateral limb served as the control arm. Repeated-measures ANOVA included all time points (baseline, cycle 3, end of treatment, 3-month follow-up, and 6-month follow-up). P values for compression vs control at each time point were derived from estimated marginal means contrasts. For total EORTC QLQ-CIPN20 scores, there were significant main effects of time ($F_{1.93,140.5}$=73.6, p<0.001, partial η^2=0.50) and arm ($F_{1,73}$=58.6, p<0.001, partial η^2=0.45), and a significant time×arm interaction ($F_{2.18,158.7}$=5.17, p=0.005, partial η^2=0.07; Greenhouse–Geisser corrected). Neuropathy scores increased over time in both arms and partially improved during follow-up. At all post-baseline assessments, compression was associated with lower CIPN20 scores compared with control.

Supplementary Table 3. Between-Limb Effect Sizes and Within-Patient Agreement for CIPN Outcomes Across Time Points

Time Point	Outcome	Cohen's d	95% CI (d)	ICC	95% CI (ICC)	p-values (ICC)
**Cycle 3**	Total CIPN	−0.24	−0.56 to 0.08	0.599	0.430–0.726	<0.001
	Total sensory	−0.21	−0.53 to 0.11	0.605	0.440–0.730	<0.001
	Hand sensory	−0.04	−0.36 to 0.28	0.350	0.087–0.556	<0.001
	Foot sensory	−0.33	−0.65 to −0.01	0.581	0.392–0.719	<0.001
	Motor	−0.14	−0.46 to 0.18	0.595	0.429–0.722	<0.001
**End of treatment**	Total CIPN	−0.21	−0.53 to 0.11	0.768	0.646–0.850	<0.001
	Total sensory	−0.16	−0.48 to 0.16	0.796	0.693–0.867	<0.001
	Hand sensory	−0.44	−0.76 to −0.12	0.753	0.402–0.881	<0.001
	Foot sensory	−0.01	−0.33 to 0.31	0.747	0.628–0.832	<0.001
	Motor	−0.36	−0.68 to −0.04	0.568	0.367–0.713	<0.001
**3-month FU**	Total CIPN	−1.54	−1.91 to −1.18	0.164	−0.083–0.414	0.001
	Total sensory	−1.54	−1.90 to −1.18	0.199	−0.090–0.477	<0.001
	Hand sensory	−1.41	−1.76 to −1.05	0.116	−0.068–0.311	0.023
	Foot sensory	−1.37	−1.73 to −1.02	0.207	−0.084–0.472	<0.001
	Motor	−2.14	−2.54 to −1.74	0.052	−0.053–0.186	0.071
**6-month FU**	Total CIPN	−1.67	−2.04 to −1.29	0.205	−0.091–0.501	<0.001
	Total sensory	−2.03	−2.43 to −1.64	0.150	−0.077–0.423	<0.001
	Hand sensory	−1.66	−2.03 to −1.28	0.164	−0.085–0.423	<0.001
	Foot sensory	−1.64	−2.01 to −1.26	0.144	−0.080–0.381	0.002
	Motor	−1.06	−1.41 to −0.72	0.204	−0.048–0.432	0.003

Abbreviations: CI (confidence interval); **CIPN** (chemotherapy-induced peripheral neuropathy); **EORTC QLQ-CIPN20** (European Organisation for Research and Treatment of Cancer Quality of Life Questionnaire–CIPN20); **FU** (follow-up); **ICC** (intraclass correlation coefficient)

Effect sizes (Cohen's d) and intraclass correlation coefficients (ICC) for total, sensory, hand sensory, foot sensory, and motor CIPN20 domains at cycle 3, end of treatment, 3-month follow-up, and 6-month follow-up. Error bars indicate 95% confidence intervals.

Supplementary Table 4. Longitudinal Transitions in Neurotoxicity After End of Treatment (CTCAE v5.0 and EORTC QLQ-CIPN20) (Panels A-B)

Panel A. CTCAE v5.0 Grade Transitions Relative to End of Treatment		
**A1. Binary transitions (≥2 = high-grade CIPN)**		
**Transition category**	**3 months after EOT (n=76)**	**6 months after EOT (n=74)**
Persistent high-grade (≥2→≥2)	27 (35.5%)	15 (20.3%)
Improved to low-grade (≥2→0–1)	20 (26.3%)	30 (40.5%)
New-onset high-grade (0–1→≥2)	0 (0.0%)	1 (1.4%)
Remained none/mild (0–1→0–1)	29 (38.2%)	28 (37.8%)
**A2. Exact grade change vs end of treatment (0–4 scale)**		
**Transition category**	**3 months after EOT (n=76)**	**6 months after EOT (n=74)**
Resolved (FU = 0)	9 (11.8%)	21 (28.4%)
Improved (FU <EOT, ≠0)	24 (31.6%)	22 (29.7%)
Persistent (FU = EOT)	29 (38.2%)	17 (23.0%)
Worsened (FU> EOT)	14 (18.4%)	14 (18.9%)

Panel B. EORTC QLQ-CIPN20 Total Score Transitions Relative to End of Treatment				
**B1. Binary transitions using CIPN20 ≥25 threshold**				
**Transition category**	**3 mo after EOT — Compression (n=76)**	**3 mo after EOT — Control (n=76)**	**6 mo after EOT — Compression (n=74)**	**6 mo after EOT — Control (n=74)**
Persistent high (≥25→≥25)	19 (25.0%)	33 (43.4%)	13 (17.6%)	21 (28.4%)
Improved (≥25→<25)	26 (34.2%)	20 (26.3%)	30 (40.5%)	30 (40.5%)
New-onset (<25→≥25)	3 (3.9%)	7 (9.2%)	2 (2.7%)	3 (4.1%)
Remained low (<25→<25)	28 (36.8%)	16 (21.1%)	29 (39.2%)	20 (27.0%)
**B2. Exact score change relative to EOT (0–100 CIPN20 transformed scale)**				
**Transition category**	**3 mo after EOT — Compression (n=76)**	**3 mo after EOT — Control (n=76)**	**6 mo after EOT — Compression (n=74)**	**6 mo after EOT — Control (n=74)**
Resolved (FU = 0)	0 (0.0%)	0 (0.0%)	0 (0.0%)	0 (0.0%)
Improved (FU <EOT, ≠0)	53 (69.7%)	50 (65.8%)	54 (73.0%)	49 (66.2%)
Persistent (FU = EOT)	0 (0.0%)	0 (0.0%)	0 (0.0%)	0 (0.0%)
Worsened (FU> EOT)	23 (30.3%)	26 (34.2%)	20 (27.0%)	25 (33.8%)

Abbreviations: CIPN, chemotherapy-induced peripheral neuropathy; CIPN20, EORTC QLQ-CIPN20; CTCAE, Common Terminology Criteria for Adverse Events; EOT, end of treatment; FU, follow-up

This table summarizes within-patient transitions in chemotherapy-induced peripheral neuropathy (CIPN) severity from end of treatment (EOT) to 3-month and 6-month follow-up. CIPN severity was categorized using EORTC QLQ-CIPN20 total score thresholds (≥25 indicating moderate–severe symptoms). Panel A reports categorical transitions based on binary CIPN stratification (persistent high, improved, new-onset, remained low). Panel B provides exact numerical change classifications relative to EOT (resolved, improved, persistent, worsened). Percentages were calculated using complete cases (n=76 at 3 months; n=74 at 6 months). These transitions illustrate the longitudinal evolution of CIPN severity during post-treatment recovery.

Supplementary Table 5. Correlation Between CTCAE Neuropathy Grade and EORTC QLQ-CIPN20 Scores Over Time

Time point	Arm	CIPN20 domain	Spearman ρ	p Value
**Baseline**	Overall	Total Sensory	0.310	0.007
		Hand sensory	0.220	0.056
		Foot sensory	0.140	0.226
		Motor	0.220	0.060
		Total	0.470	<0.001
**Cycle 3**	Compression	Total Sensory	0.830	<0.001
		Hand sensory	0.825	<0.001
		Foot sensory	0.833	<0.001

Time point	Arm	CIPN20 domain	Spearman ρ	p Value
		Motor	0.830	<0.001
		Total	0.830	<0.001
	Control	Total Sensory	0.700	<0.001
		Hand sensory	0.706	<0.001
		Foot sensory	0.696	<0.001
		Motor	0.700	<0.001
		Total	0.710	<0.001
**End of Treatment**	Compression	Total Sensory	0.850	<0.001
		Hand sensory	0.833	<0.001
		Foot sensory	0.850	<0.001
		Motor	0.860	<0.001
		Total	0.860	<0.001
	Control	Total Sensory	0.830	<0.001
		Hand sensory	0.833	<0.001
		Foot sensory	0.816	<0.001
		Motor	0.740	<0.001
		Total	0.820	<0.001
**3-month Follow-up**	Compression	Total Sensory	0.390	0.001
		Hand sensory	0.386	0.001
		Foot sensory	0.343	0.002
		Motor	0.240	0.040
		Total	0.330	0.004
	Control	Total Sensory	0.520	<0.001
		Hand sensory	0.379	0.001
		Foot sensory	0.519	<0.001
		Motor	0.290	0.010
		Total	0.460	0.003
**6-month Follow-up**[1]	Compression	Total Sensory	0.360	0.002
		Hand sensory	0.358	0.002
		Foot sensory	0.348	0.002
		Motor	0.360	0.002
		Total	0.380	0.001
	Control	Total Sensory	0.410	<0.001
		Hand sensory	0.250	0.032
		Foot sensory	0.232	0.047
		Motor	0.360	0.001
		Total	0.430	<0.001

Abbreviations: CIPN, chemotherapy-induced peripheral neuropathy; **CIPN20**, EORTC QLQ-CIPN20; **CTCAE**, Common Terminology Criteria for Adverse Events; **EORTC**, European Organisation for Research and Treatment of Cancer; **ρ**, Spearman correlation coefficient.

[1] Two patients missing at 6-month follow-up.

Spearman correlation coefficients (ρ) between clinician-graded neuropathy severity (CTCAE grade ≥2) and patient-reported CIPN symptoms (EORTC QLQ-CIPN20 sensory, motor, and total scores) across study time points. Higher coefficients indicate stronger monotonic association between CTCAE and CIPN20 scores. Analyses were performed separately for compression and control limbs where applicable. P values are two-sided.

Supplementary Table 6. Agreement Between Clinician-Graded Neuropathy (CTCAE ≥2) and Patient-Reported CIPN Severity (CIPN20 ≥25) Over Time

Time point	Arm	Cohen's κ	95% CI	p value	Percent agreement, %	PABAK	n/N
**Cycle 3**	Compression	**0.819**	0.682–0.956	<.001	89.5	**0.789**	68/76
	Control	**0.390**	0.216–0.564	<.001	64.5	**0.289**	49/76
**End of treatment**	Compression	**0.725**	0.566–0.884	<.001	78.9	**0.579**	60/76
	Control	**0.768**	0.619–0.917	<.001	76.3	**0.526**	58/76
**3 months after EOT**	Compression	**0.312**	0.128–0.496	.002	64.5	**0.289**	49/76
	Control	**0.352**	0.152–0.552	.001	71.1	**0.421**	54/76
**6 months after EOT**	Compression	**0.201**	0.034–0.368	.024	58.1	**0.162**	43/74
	Control	**0.275**	0.045–0.505	.018	66.2	**0.324**	49/74

Abbreviations: CIPN, chemotherapy-induced peripheral neuropathy; CIPN20, EORTC QLQ-CIPN20; CTCAE, Common Terminology Criteria for Adverse Events; EORTC, European Organisation for Research and Treatment of Cancer; EOT, end of treatment; PABAK, prevalence-adjusted bias-adjusted kappa.

Cohen's κ values, 95% confidence intervals, percent agreement, and PABAK are shown to demonstrate concordance between clinician-assessed neuropathy and patient-reported symptoms at treatment and follow-up visits.

Percent agreement was calculated as the proportion of concordant classifications between CTCAE grade ≥2 and CIPN20 ≥25. PABAK (prevalence-adjusted bias-adjusted kappa) was computed as **PABAK = (2 × Percent Agreement) − 1***, which provides a prevalence-robust measure of agreement when category frequencies are unbalanced.*

Supplementary Table 7. Diagnostic performance and ROC analysis of EORTC QLQ-CIPN20 scores against CTCAE v5.0 grading at Cycle 3, End of Treatment (EOT), and follow-up assessments (3-mo and 6-mo)

Domain	Arm	Time point	AUC (95% CI)	p-value	Sensitivity	Specificity	PPV	NPV	Accuracy
**Sensory CIPN**	Compression	Cycle 3	0.922 (0.861–0.983)	<0.001	0.90	0.71	0.74	0.89	0.82
	Control	Cycle 3	0.906 (0.835–0.976)	<0.001	0.86	0.76	0.77	0.85	0.81
	Compression	EOT	0.965 (0.928–1.000)	<0.001	0.96	0.83	0.82	0.96	0.89
	Control	EOT	0.977 (0.952–1.000)	<0.001	0.94	0.88	0.88	0.94	0.91
	Compression	3-mo follow-up	0.748 (0.608-0.888)	<0.001	0.70	0.90	0.79	0.85	0.83
	Control	3-mo follow-up	0.827 (0.721–0.933)	<0.001	0.82	0.80	0.69	0.89	0.80
	Compression	6-mo follow-up	0.836 (0.694–0.978)	<0.001	0.81	0.79	0.52	0.94	0.80
	Control	6-mo follow-up	0.821 (0.689–0.952)	<0.001	0.82	0.79	0.51	0.93	0.81
**Motor CIPN**	Compression	Cycle 3	0.782 (0.666–0.898)	<0.001	0.57	0.84	0.72	0.74	0.73
	Control	Cycle 3	0.794 (0.681–0.908)	<0.001	0.62	0.79	0.68	0.74	0.71
	Compression	EOT	0.807 (0.711–0.903)	<0.001	0.60	0.82	0.71	0.74	0.72
	Control	EOT	0.840 (0.752–0.928)	<0.001	0.65	0.80	0.69	0.77	0.73
	Compression	3-mo follow-up	0.675 (0.523–0.826)	0.012	0.67	0.45	0.40	0.71	0.53
	Control	3-mo follow-up	0.671 (0.540–0.803)	0.014	0.82	0.37	0.42	0.78	0.53
	Compression	6-mo follow-up	0.808 (0.673–0.943)	<0.001	0.88	0.59	0.37	0.94	0.65
	Control	6-mo follow-up	0.789 (0.637–0.941)	<0.001	0.81	0.78	0.50	0.94	0.78
**Total CIPN**	Compression	Cycle 3	0.972 (0.942–1.000)	<0.001	0.95	0.88	0.90	0.94	0.92
	Control	Cycle 3	0.873 (0.790–0.956)	<0.001	0.85	0.81	0.83	0.84	0.84
	Compression	EOT	0.960 (0.923–0.998)	<0.001	0.93	0.86	0.87	0.92	0.90
	Control	EOT	0.950 (0.891–1.000)	<0.001	0.92	0.84	0.85	0.91	0.88
	Compression	3-mo follow-up	0.717 (0.567–0.868)	0.002	0.70	0.92	0.83	0.85	0.84
	Control	3-mo follow-up	0.795 (0.684–0.905)	<0.001	0.89	0.53	0.51	0.90	0.66
	Compression	6-mo follow-up	0.824 (0.672–0.975)	<0.001	0.81	0.86	0.62	0.94	0.85
	Control	6-mo follow-up	0.832 (0.701–0.964)	<0.001	0.88	0.83	0.58	0.96	0.84

Abbreviations: AUC, area under the curve; CIPN, chemotherapy-induced peripheral neuropathy; CTCAE, Common Terminology Criteria for Adverse Events (version 5.0); EOT, end of treatment; NPV, negative predictive value; PPV, positive predictive value.

Supplementary Table 8. Univariable analysis of factors associated with moderate-to-severe CIPN at end of treatment

Variable (n=76)	OR (95% CI)	p-value
Age (per 10 years)	**1.71 (1.20–2.44)**	**0.003**
BMI (per 5 kg/m²)	1.21 (0.94–1.56)	0.142
HbA1c (per 1% increase)	**1.77 (1.18–2.65)**	**0.006**
Magnesium (per 0.1 mg/dL increase)	**0.90 (0.81–0.99)**	**0.031**
Vitamin B12 (per 100 pg/mL increase)	**0.72 (0.58–0.88)**	**0.003**
Vitamin D (per 5 ng/mL increase)	**0.63 (0.51–0.77)**	**<0.001**
CAR (per 0.1 unit increase)	**0.96 (0.91–1.00)**	**0.070**
Cumulative Taxane Dose (per 100 mg/m² increase)	**1.10 (0.99–1.21)**	**0.094**
ECOG-PS (2 vs 0–1)	2.45 (0.61–7.42)	0.107
Smoking History (present vs absent)	1.22 (0.45–3.30)	0.692
Alcohol History (present vs absent)	2.93 (0.31–27.53)	0.328
Metformin Use (yes vs no)	**2.74 (0.98–7.66)**	**0.050**
mCCI (cut-off:8)	1.74 (0.68–4.45)	0.247
Baseline CA-125 (U/mL) (cut-off:35)	2.79 (0.35–7.38)	0.136

Variable (n=76)	OR (95% CI)	p-value
Disease Stage (III–IV vs I–II)	1.33 (0.53–3.34)	0.550
Primary Diagnosis (endometrial vs ovarian cancer)	1.45 (0.68–3.13)	0.133

Abbreviations: BMI, body mass index; CAR, C-reactive protein–to–albumin ratio; CI, confidence interval; CIPN, chemotherapy-induced peripheral neuropathy; ECOG, Eastern Cooperative Oncology Group; OR, odds ratio.

Notes: Univariable analyses were performed at the limb level using end-of-treatment outcomes. Continuous variables were modeled per clinically meaningful unit. Compression was applied using a within-patient paired design and was therefore evaluated exclusively in the primary generalized estimating equation (GEE) model, not in univariable analyses.

Cycle 3 CIPN scores were not included in univariable analyses of risk factors because they were measured after initiation of compression and may represent early manifestations of the outcome rather than baseline predictors.

Supplementary Figure 1. Measurement Methods for Hand, Ankle, and Calf Circumference With Corresponding Sizing and Compression Classifications

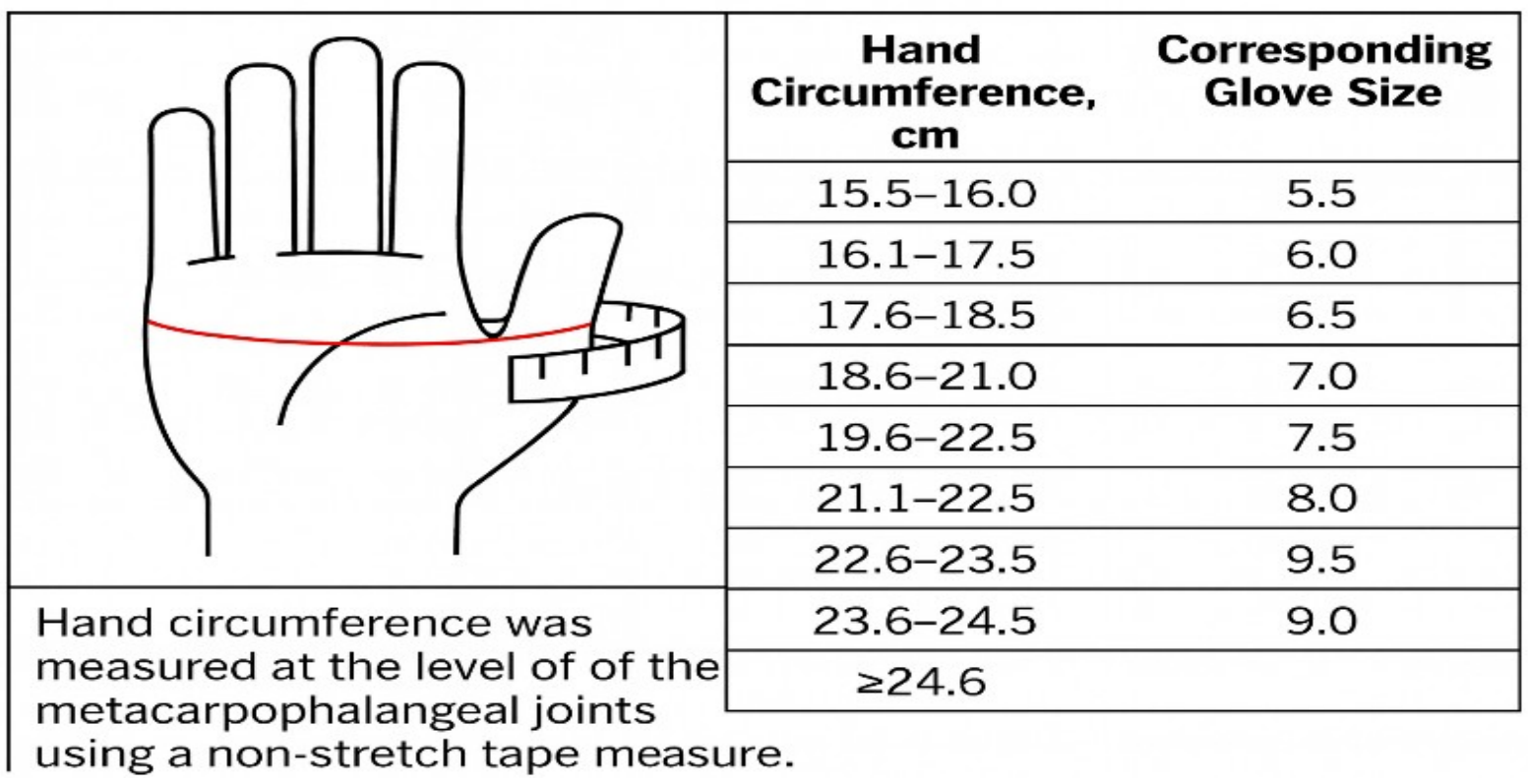

Hand Circumference, cm	Corresponding Glove Size
15.5–16.0	5.5
16.1–17.5	6.0
17.6–18.5	6.5
18.6–21.0	7.0
19.6–22.5	7.5
21.1–22.5	8.0
22.6–23.5	9.5
23.6–24.5	9.0
≥24.6	

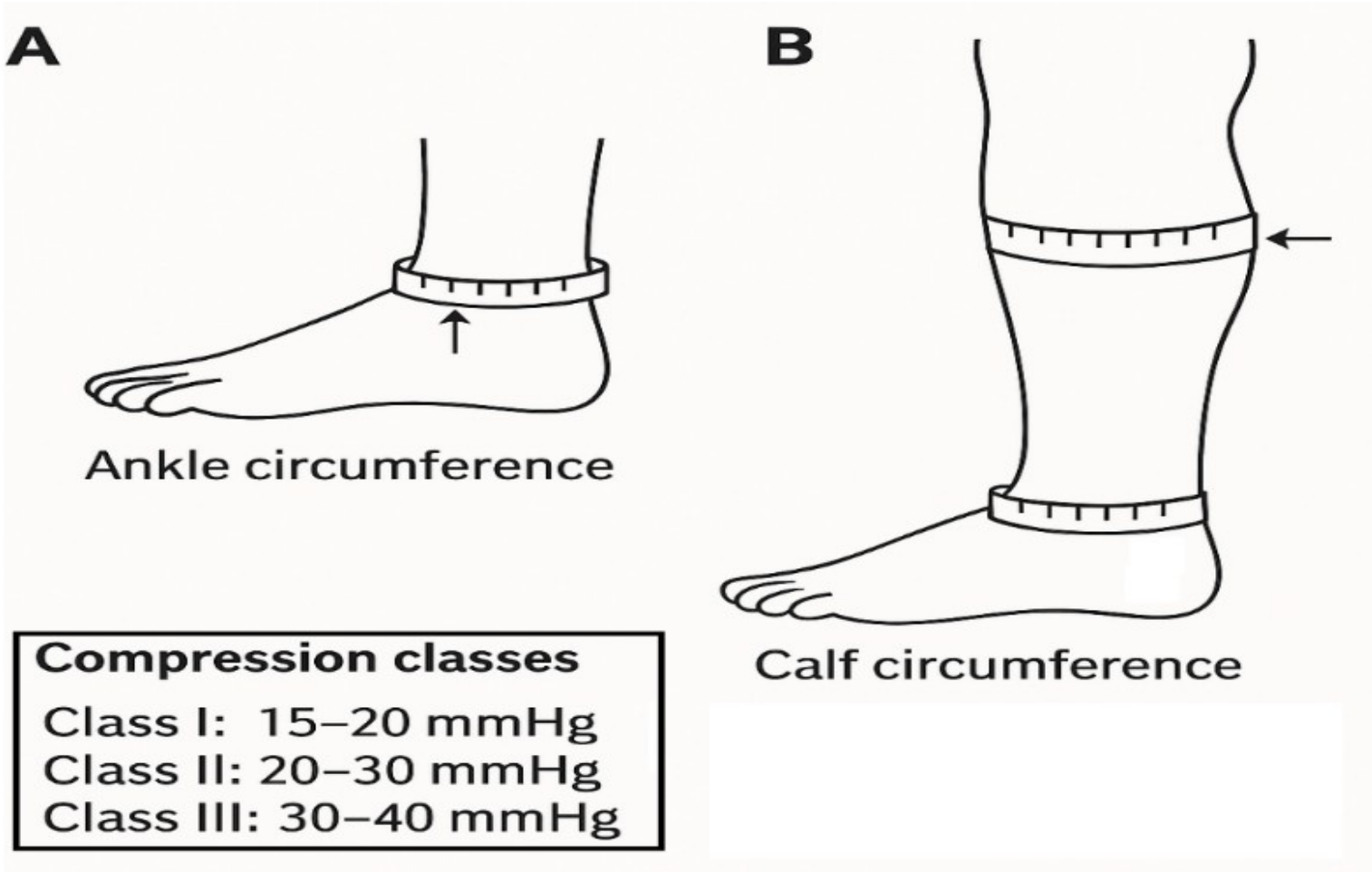

Abbreviations: cm, centimeter; mmHg, millimeters of mercury.

Hand circumference was measured at the level of the metacarpophalangeal joints using a nonstretch tape measure to determine appropriate glove size. Ankle and calf circumferences were measured to classify compression therapy levels for lower extremity garments. All measurements were performed bilaterally at baseline prior to initiation of chemotherapy by trained clinical staff using standardized procedures. Corresponding sizing intervals and compression classes are shown in the accompanying tables.

Supplementary Figure 2. Longitudinal Transitions of Neuropathy Severity Across Time Points Based on CTCAE and CIPN20 Scores (Panels A-C)

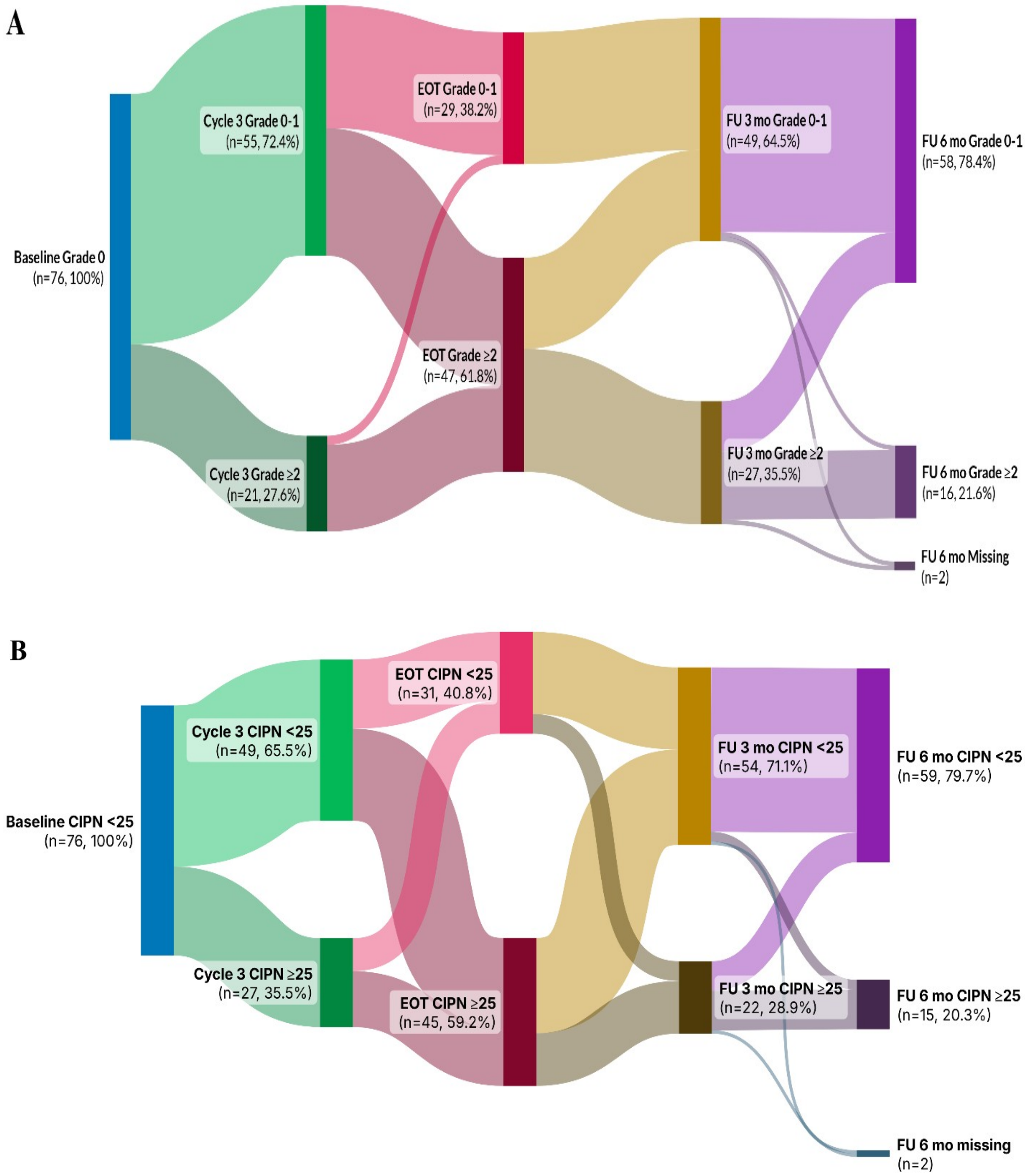

A
Baseline Grade 0
(n=76, 100%)
Cycle 3 Grade 0-1
(n=55, 72.4%)
Cycle 3 Grade ≥2
(n=21, 27.6%)
EOT Grade 0-1
(n=29, 38.2%)
EOT Grade ≥2
(n=47, 61.8%)
FU 3 mo Grade 0-1
(n=49, 64.5%)
FU 3 mo Grade ≥2
(n=27, 35.5%)
FU 6 mo Grade 0-1
(n=58, 78.4%)
FU 6 mo Grade ≥2
(n=16, 21.6%)
FU 6 mo Missing
(n=2)
B
Baseline CIPN <25
(n=76, 100%)
Cycle 3 CIPN <25
(n=49, 65.5%)
Cycle 3 CIPN ≥25
(n=27, 35.5%)
EOT CIPN <25
(n=31, 40.8%)
EOT CIPN ≥25
(n=45, 59.2%)
FU 3 mo CIPN <25
(n=54, 71.1%)
FU 3 mo CIPN ≥25
(n=22, 28.9%)
FU 6 mo CIPN <25
(n=59, 79.7%)
FU 6 mo CIPN ≥25
(n=15, 20.3%)
FU 6 mo missing
(n=2)

C

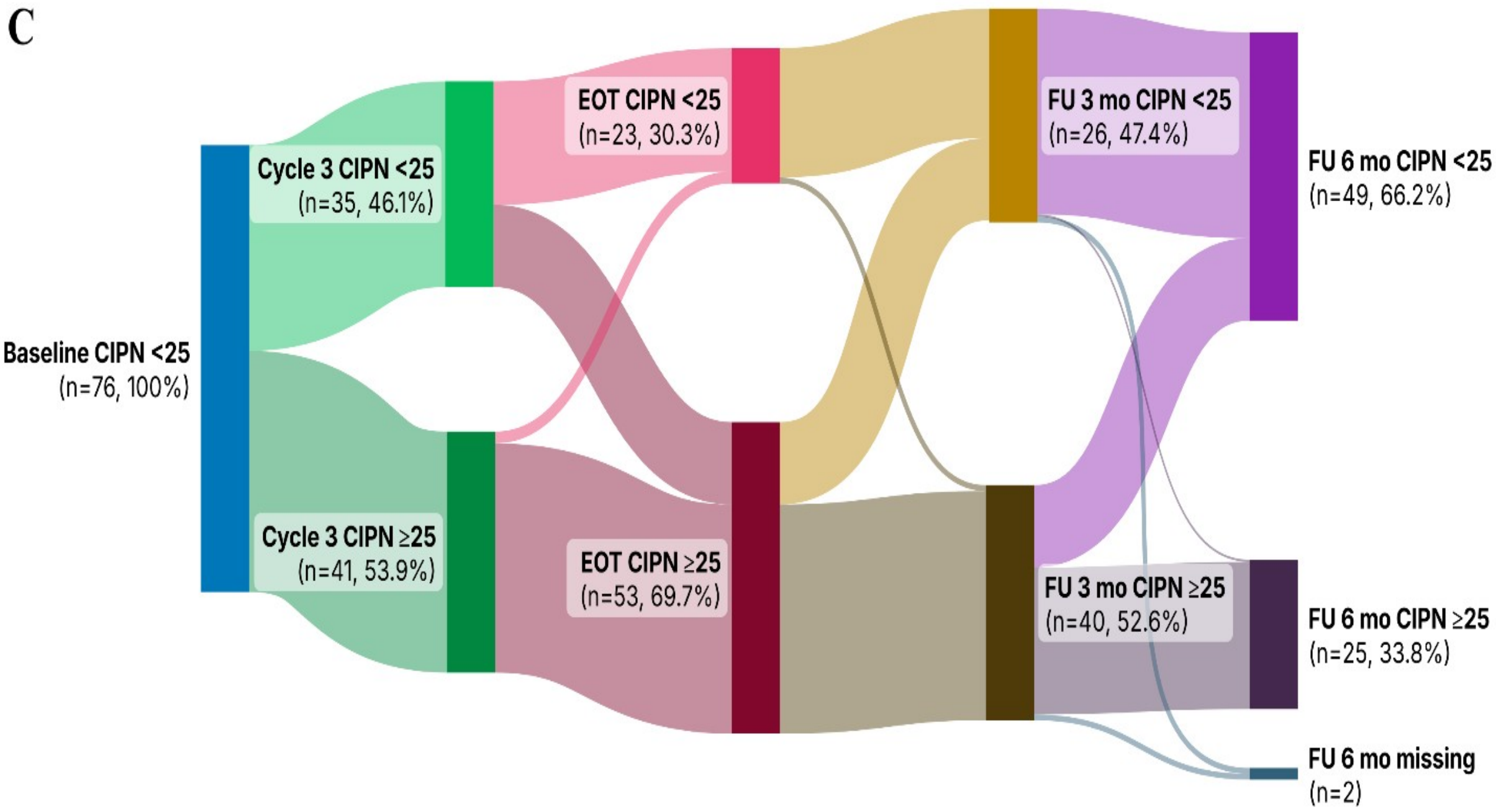

Abbreviations: CIPN, chemotherapy-induced peripheral neuropathy; CIPN20, EORTC QLQ-CIPN20; CTCAE, Common Terminology Criteria for Adverse Events; EOT, end of treatment; FU, follow-up; mo, Months.

(A) Transitions in clinician-graded neuropathy (CTCAE v5.0) from baseline through cycle 3, end of treatment (EOT), 3-month follow-up, and 6-month follow-up (p<0.001; Cramér's V = 0.44). At 6 months, assessments were not available for 2 patients due to death.
(B) Sankey diagram illustrating transitions in patient-reported total CIPN severity (CIPN20 ≥25 vs <25) for the **compression arm (**(p<0.001; Cramér's V = 0.47).
(C) Corresponding transitions in patient-reported total CIPN severity for the **control arm** (p<0.001; Cramér's V = 0.48). Flow widths are proportional to the number of patients exhibiting a given transition between time points.

Supplementary Figure 3. Receiver Operating Characteristic Curves for EORTC QLQ-CIPN20 Sensory, Motor, and Total Scores Against CTCAE v5.0 Neuropathy Grading (Panels A–D)

A

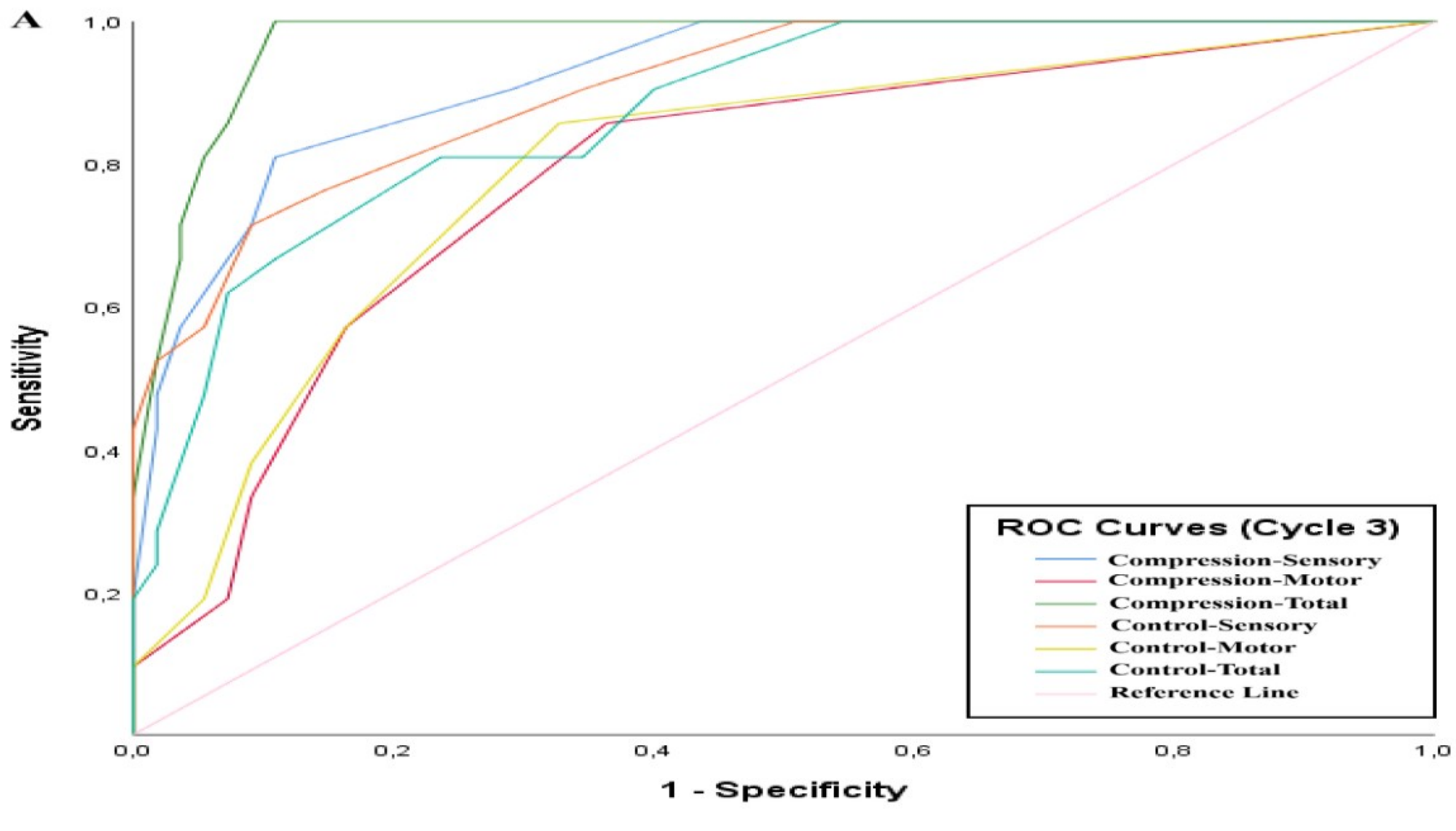

B

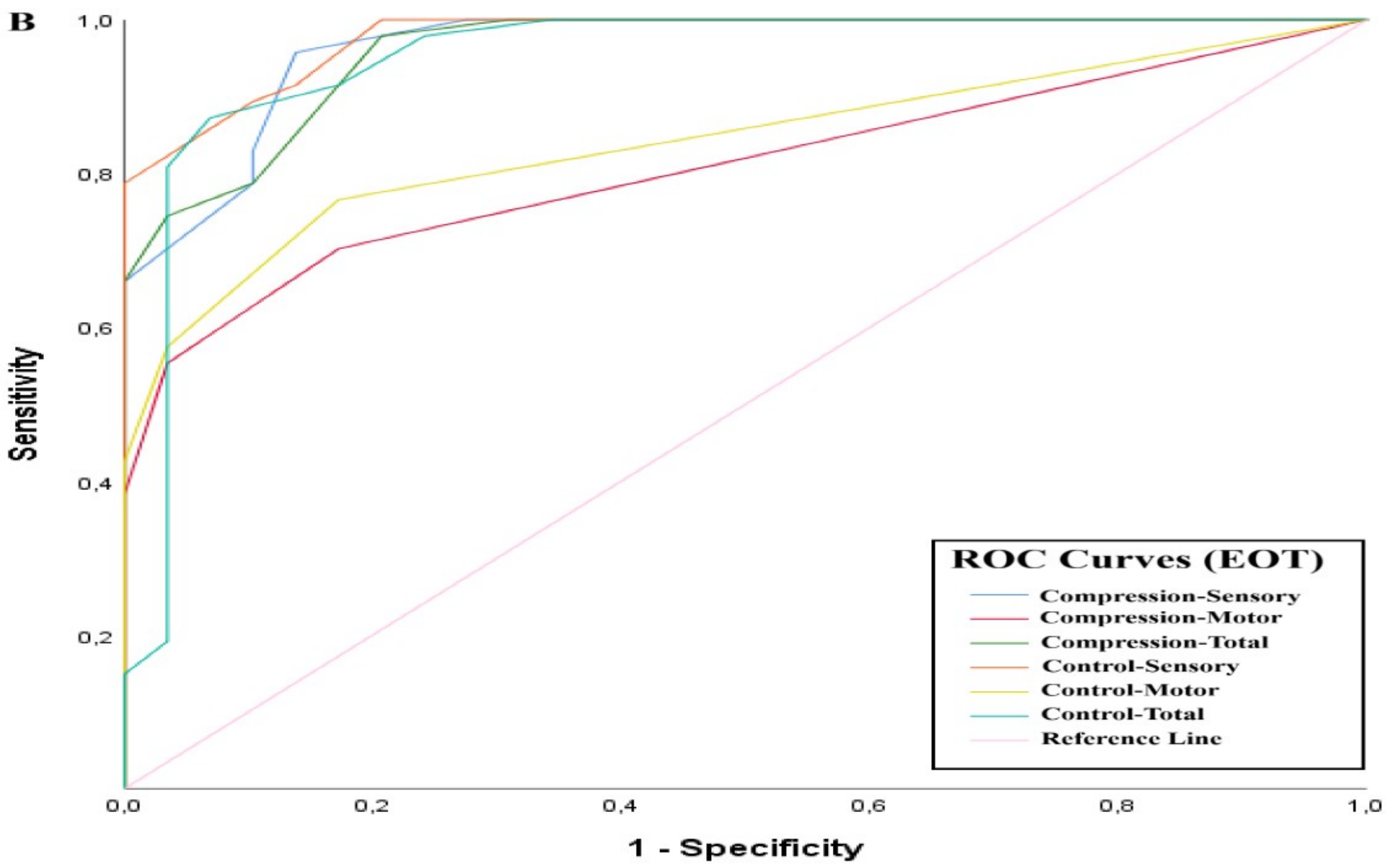
1,0
0,8
0,6
0,4
0,2
Sensitivity
0,0
0,2
0,4
0,6
0,8
1,0
1 - Specificity
ROC Curves (EOT)
Compression-Sensory
Compression-Motor
Compression-Total
Control-Sensory
Control-Motor
Control-Total
Reference Line

C

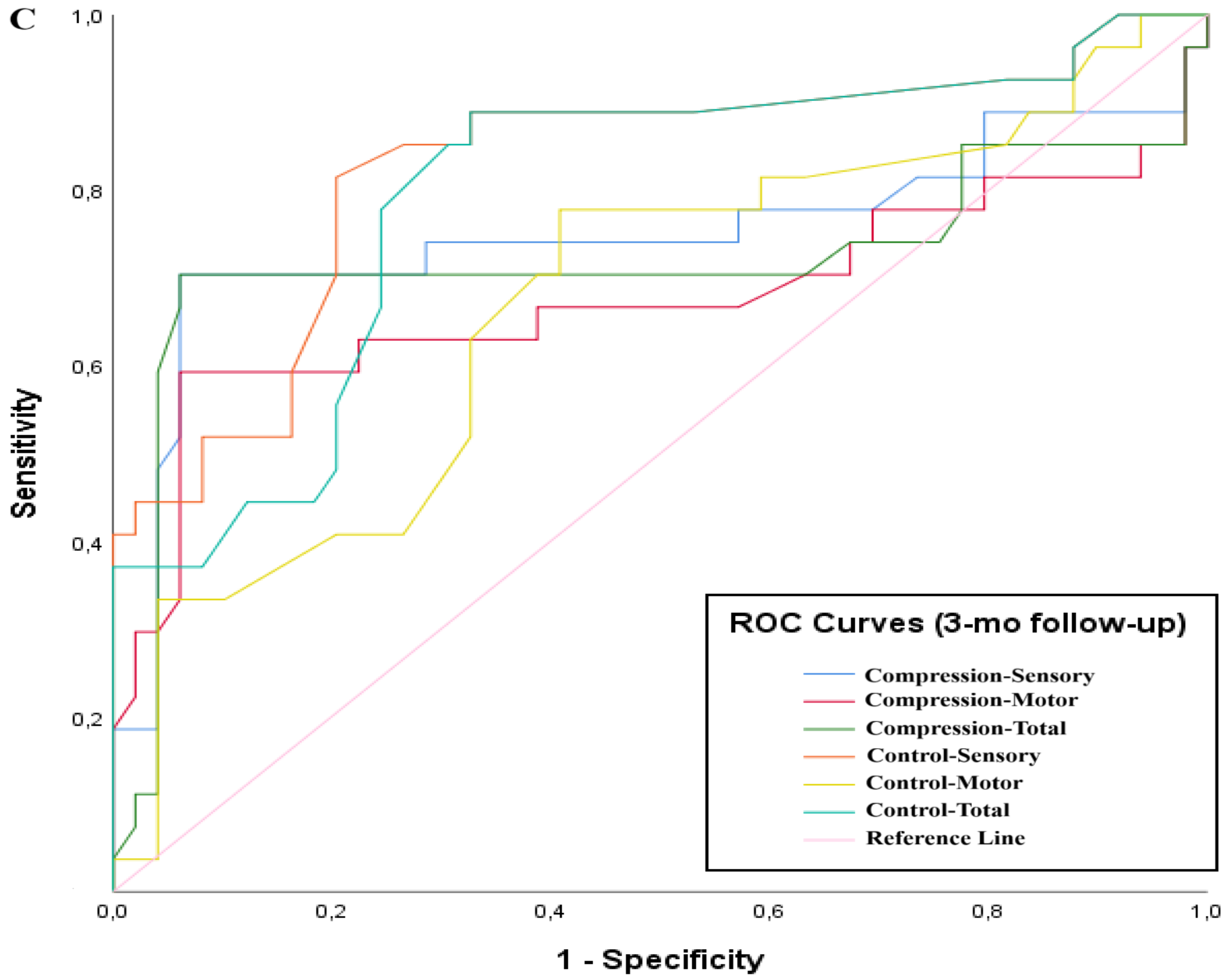
1,0
0,8
0,6
0,4
0,2
Sensitivity
0,0
0,2
0,4
0,6
0,8
1,0
1 - Specificity
ROC Curves (3-mo follow-up)
Compression-Sensory
Compression-Motor
Compression-Total
Control-Sensory
Control-Motor
Control-Total
Reference Line

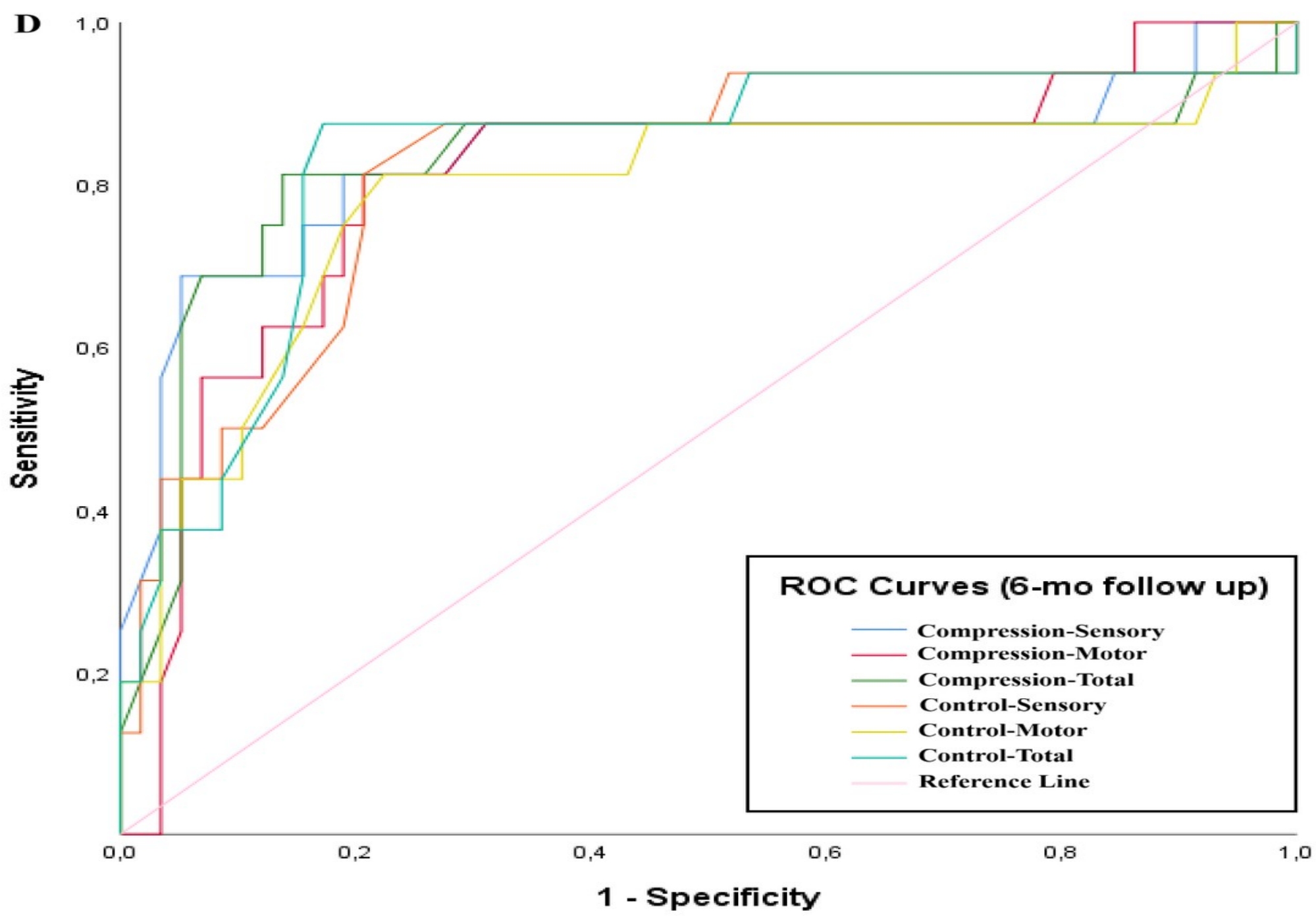

Abbreviations: CIPN, chemotherapy-induced peripheral neuropathy; CIPN20, EORTC QLQ-CIPN20; CTCAE, Common Terminology Criteria for Adverse Events; EORTC, European Organisation for Research and Treatment of Cancer; EOT, end of treatment; ROC, receiver operating characteristic.

(A) Cycle 3; **(B)** End of Treatment (EOT); **(C)** 3-month follow-up; **(D)** 6-month follow-up.
Each panel displays ROC curves for sensory, motor, and total CIPN20 domain scores in the compression and control limbs, using CTCAE grade ≥2 neuropathy as the reference standard. Curves illustrate the discriminative ability of each CIPN20 domain in detecting moderate–severe neuropathy at matched time points.

Supplementary Figure 4. Variable Importance Derived From Random Forest Models Predicting Moderate-to-Severe CIPN at End of Treatment

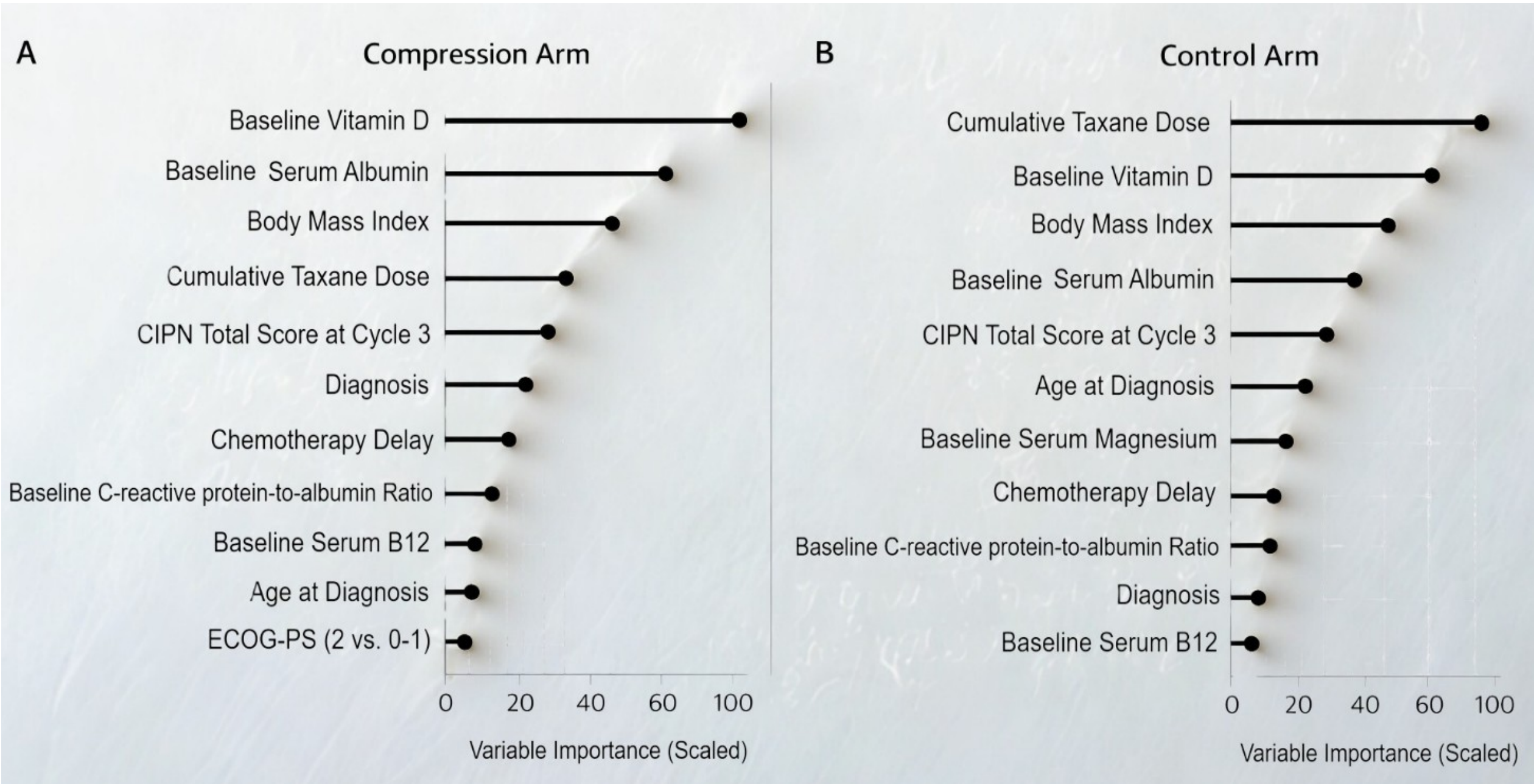

Abbreviations: BMI, body mass index; CIPN, chemotherapy-induced peripheral neuropathy; ECOG, Eastern Cooperative Oncology Group; HbA1c, hemoglobin A1c

Plots display the scaled variable importance scores for the top predictors of neuropathy in the (A) compression limb and (B) control limb. Features are ranked based on the mean decrease in Gini impurity, with scores scaled relative to the most influential predictor (set to 100) within each model.

Supplementary Figure 5. Receiver Operating Characteristic Curves for Random Forest Models Predicting Moderate-to-Severe CIPN at End of Treatment (Panels A-B)

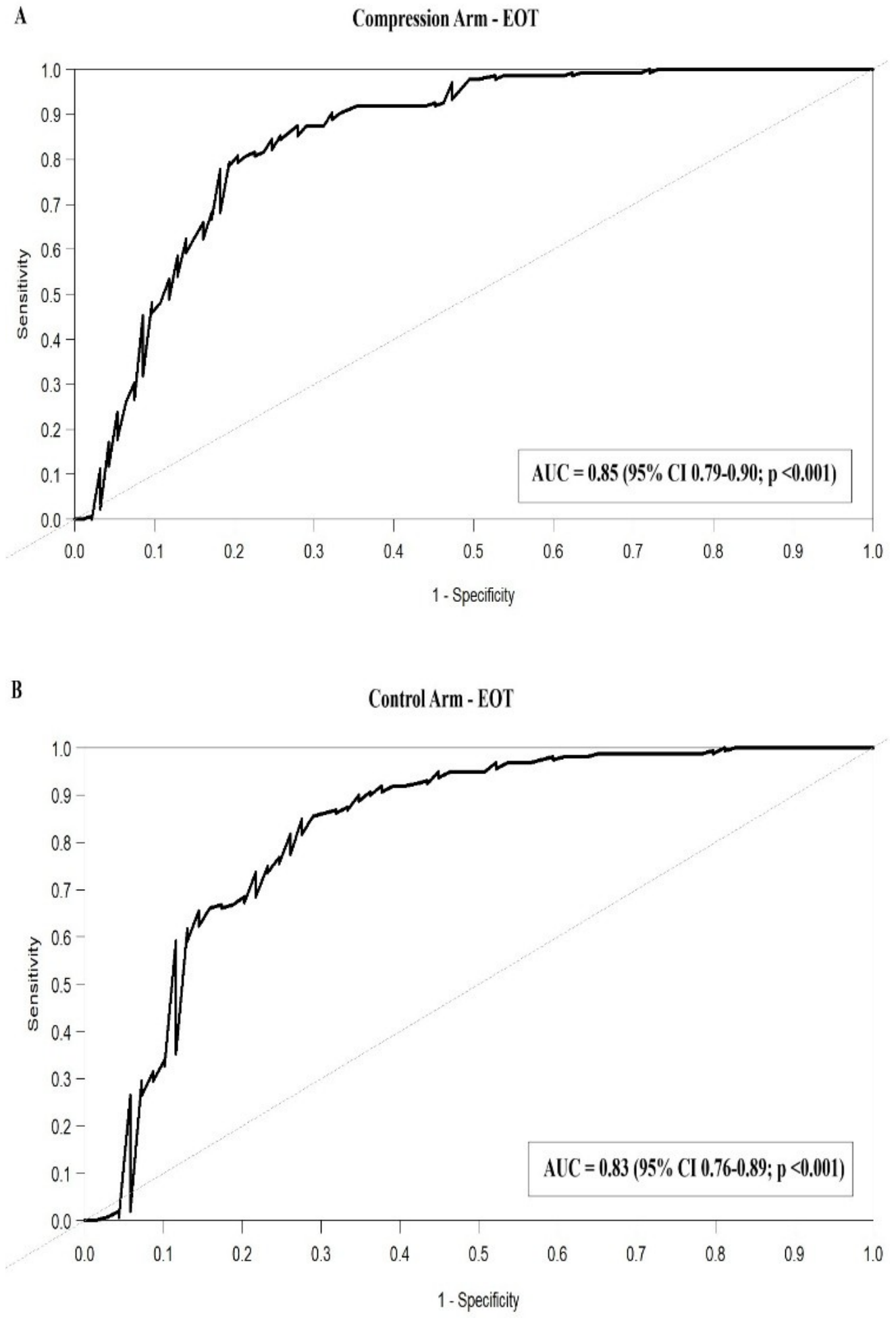

Abbreviations: AUC, area under the curve; CIPN, chemotherapy-induced peripheral neuropathy; CI, confidence interval; MCC, Matthews correlation coefficient; ROC, receiver operating characteristic.

Panel A shows the ROC curve for the compression-arm model and Panel B for the control-arm model. The AUC and 95% CI are indicated within each panel. Model thresholds, sensitivity, specificity, accuracy, balanced accuracy, and MCC values are reported in the text.

EOT – Compression (ROC): AUC = 0.85 (95% CI 0.79–0.90; p<0.001); threshold = 0.569; sensitivity = 0.81; specificity = 0.80; accuracy = 0.80; balanced accuracy = 0.81; MCC = 0.60. EOT – Control (ROC): AUC = 0.83 (95% CI 0.76–0.89; p<0.001); threshold = 0.625; sensitivity = 0.85; specificity = 0.72; accuracy = 0.81; balanced accuracy = 0.79; MCC = 0.58. Between-model comparison (panel A): DeLong comparison (compression vs control): p = 0.666.

Appendix 1

TREND Statement Checklist

TREND Statement Checklist

Paper Section/ Topic	Item No	Descriptor	Reported? ✓	Pg #
**Title and Abstract**				
Title and Abstract	1	• Information on how unit were allocated to interventions	yes	1
		• Structured abstract recommended	yes	2
		• Information on target population or study sample	yes	2
**Introduction**				
Background	2	• Scientific background and explanation of rationale	yes	3
		• Theories used in designing behavioral interventions	yes	3
**Methods**				
Participants	3	• Eligibility criteria for participants, including criteria at different levels in recruitment/sampling plan (e.g., cities, clinics, subjects)	yes	4-5
		• Method of recruitment (e.g., referral, self-selection), including the sampling method if a systematic sampling plan was implemented	yes	4-5
		• Recruitment setting	yes	4-5
		• Settings and locations where the data were collected	yes	4-5
Interventions	4	• Details of the interventions intended for each study condition and how and when they were actually administered, specifically including:	yes	5-6
		○ Content: what was given?		
		○ Delivery method: how was the content given?		
		○ Unit of delivery: how were the subjects grouped during delivery?		
		○ Deliverer: who delivered the intervention?		
		○ Setting: where was the intervention delivered?		
		○ Exposure quantity and duration: how many sessions or episodes or events were intended to be delivered? How long were they intended to last?		
		○ Time span: how long was it intended to take to deliver the intervention to each unit?		
		○ Activities to increase compliance or adherence (e.g., incentives)		
Objectives	5	• Specific objectives and hypotheses	yes	3
Outcomes	6	• Clearly defined primary and secondary outcome measures	yes	4-5
		• Methods used to collect data and any methods used to enhance the quality of measurements		
		• Information on validated instruments such as psychometric and biometric properties		
Sample Size	7	• How sample size was determined and, when applicable, explanation of any interim analyses and stopping rules	yes	3
Assignment Method	8	• Unit of assignment (the unit being assigned to study condition, e.g., individual, group, community)	yes	3
		• Method used to assign units to study conditions, including details of any restriction (e.g., blocking, stratification, minimization)	yes	3
		• Inclusion of aspects employed to help minimize potential bias induced due to non-randomization (e.g., matching)	yes	3

TREND Statement Checklist

Blinding (masking)	9	• Whether or not participants, those administering the interventions, and those assessing the outcomes were blinded to study condition assignment; if so, statement regarding how the blinding was accomplished and how it was assessed.	no	N/A
Unit of Analysis	10	• Description of the smallest unit that is being analyzed to assess intervention effects (e.g., individual, group, or community)	yes	5-6
		• If the unit of analysis differs from the unit of assignment, the analytical method used to account for this (e.g., adjusting the standard error estimates by the design effect or using multilevel analysis)	yes	5-6
Statistical Methods	11	• Statistical methods used to compare study groups for primary methods outcome(s), including complex methods of correlated data	yes	5-6
		• Statistical methods used for additional analyses, such as a subgroup analyses and adjusted analysis	yes	5-6
		• Methods for imputing missing data, if used	yes	5-6
		• Statistical software or programs used	yes	5-6
**Results**				
Participant flow	12	• Flow of participants through each stage of the study: enrollment, assignment, allocation, and intervention exposure, follow-up, analysis (a diagram is strongly recommended)	yes	fig 1
		○ Enrollment: the numbers of participants screened for eligibility, found to be eligible or not eligible, declined to be enrolled, and enrolled in the study		
		○ Assignment: the numbers of participants assigned to a study condition		
		○ Allocation and intervention exposure: the number of participants assigned to each study condition and the number of participants who received each intervention		
		○ Follow-up: the number of participants who completed the follow-up or did not complete the follow-up (i.e., lost to follow-up), by study condition		
		○ Analysis: the number of participants included in or excluded from the main analysis, by study condition		
		• Description of protocol deviations from study as planned, along with reasons		
Recruitment	13	• Dates defining the periods of recruitment and follow-up	yes	6
Baseline Data	14	• Baseline demographic and clinical characteristics of participants in each study condition	yes	table 1
		• Baseline characteristics for each study condition relevant to specific disease prevention research		
		• Baseline comparisons of those lost to follow-up and those retained, overall and by study condition		
		• Comparison between study population at baseline and target population of interest		
Baseline equivalence	15	• Data on study group equivalence at baseline and statistical methods used to control for baseline differences	yes	table 1

TREND Statement Checklist

Numbers analyzed	16	• Number of participants (denominator) included in each analysis for each study condition, particularly when the denominators change for different outcomes; statement of the results in absolute numbers when feasible	yes	figure 1
		• Indication of whether the analysis strategy was "intention to treat" or, if not, description of how non-compliers were treated in the analyses	yes	figure 1
Outcomes and estimation	17	• For each primary and secondary outcome, a summary of results for each estimation study condition, and the estimated effect size and a confidence interval to indicate the precision	yes	figure 2-4
		• Inclusion of null and negative findings	yes	figure 2-4
		• Inclusion of results from testing pre-specified causal pathways through which the intervention was intended to operate, if any	yes	figure 2-4
Ancillary analyses	18	• Summary of other analyses performed, including subgroup or restricted analyses, indicating which are pre-specified or exploratory	yes	supplementary file
Adverse events	19	• Summary of all important adverse events or unintended effects in each study condition (including summary measures, effect size estimates, and confidence intervals)	yes	figure 1
**DISCUSSION**				
Interpretation	20	• Interpretation of the results, taking into account study hypotheses, sources of potential bias, imprecision of measures, multiplicative analyses, and other limitations or weaknesses of the study	yes	7-8
		• Discussion of results taking into account the mechanism by which the intervention was intended to work (causal pathways) or alternative mechanisms or explanations	yes	7-8
		• Discussion of the success of and barriers to implementing the intervention, fidelity of implementation	yes	7-8
		• Discussion of research, programmatic, or policy implications	yes	7-8
Generalizability	21	• Generalizability (external validity) of the trial findings, taking into account the study population, the characteristics of the intervention, length of follow-up, incentives, compliance rates, specific sites/settings involved in the study, and other contextual issues	yes	8
Overall Evidence	22	• General interpretation of the results in the context of current evidence and current theory	yes	8

From: Des Jarlais, D. C., Lyles, C., Crepaz, N., & the Trend Group (2004). Improving the reporting quality of nonrandomized evaluations of behavioral and public health interventions: The TREND statement. *American Journal of Public Health*, 94, 361-366. For more information, visit: http://www.cdc.gov/trendstatement/
